# Risk of biodiversity collapse under climate change in the Afro-Arabian region

**DOI:** 10.1038/s41598-018-37851-6

**Published:** 2019-01-30

**Authors:** Alaaeldin Soultan, Martin Wikelski, Kamran Safi

**Affiliations:** 1 Max Planck Institute for Ornithology, Department of Migration and Immuno-ecology, Am Obstberg 1, 78315 Radolfzell, Germany; 2 University of Konstanz, Department of Biology, Universitätsstraße 10, 78464 Konstanz, Germany

## Abstract

For 107 endemic mammal species in the Afro-Arabian region, Sahara-Sahel and Arabian Desert, we used ensemble species distribution models to: (1) identify the hotspot areas for conservation, (2) assess the potential impact of the projected climate change on the distribution of the focal species, and (3) assign IUCN threat categories for the focal species according to the predicted changes in their potential distribution range. We identified two main hotspot areas for endemic mammals: the Sinai and its surrounding coastal area in the East, and the Mediterranean Coast around Morocco in the West. Alarmingly, our results indicate that about 17% of the endemic mammals in the Afro-Arabian region under the current climate change scenarios could go extinct before 2050. Overall, a substantial number of the endemic species will change from the IUCN threat category “Least Concern” to “Critically Endangered” or “Extinct” in the coming decades. Accordingly, we call for implementing an urgent proactive conservation action for these endemic species, particularly those that face a high risk of extinction in the next few years. The results of our study provide conservation managers and practitioners with the required information for implementing an effective conservation plan to protect the biodiversity of the Afro-Arabian region.

## Introduction

The unprecedented loss of biodiversity, with an estimated extinction of a species every 20 minutes^1^, indicates that a “sixth mass extinction” is under way^1–4^. There is now overwhelming evidence that both climate change and habitat fragmentation are together responsible for this exceptional biodiversity loss^5^, with further accelerated extinction predicted during the next decades^2,5^. Unless we implement effective conservation measures immediately about two-thirds of the vertebrate populations could vanish by 2020^3^. Accordingly, the Convention on Biological Diversity listed strategic goals to forestall the ongoing loss of biodiversity^6^. Assessing the current and future biodiversity status and future trends are imperative to achieve these goals^7,8^.

Previous empirical studies show that species’ ecological traits influence their response to climate change and that response varies across species^9,10^. Traits that may increase risk of extinction because of climate change include: (1) narrow distribution ranges; (2) limited dispersal ability; (3) high level of specialisation, and (4) low reproductive performance^11^. Narrow-ranged species, tend to possess most of these traits, and are therefore more vulnerable to climate change than other species^11,12^. Accordingly, assessing the potential impact of climate change on biodiversity patterns and species distribution is essential to determine the most vulnerable species and areas, which would allow for prioritising conservation efforts. Several assessments at global and continental scales^12–16^ have been carried out; however, effective conservation plans require information at the more local scale of biomes or ecoregions^17,18^.

**Figure 1:**
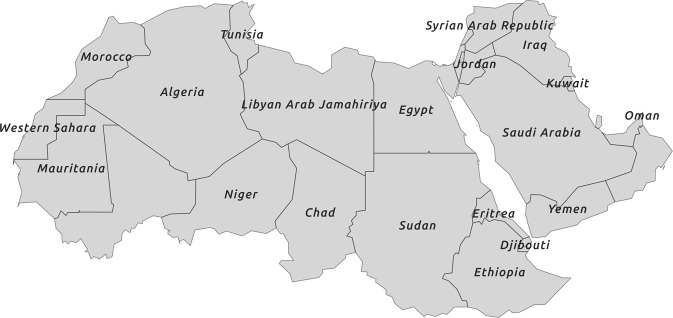
The geographic location of the Afro-Arabian region.

The warm desert biome has been neglected in conservation ecology^2,8,19^. Yet, the warm desert biome encompassing the Sahara and the Arabian Desert (Fig. 1; henceforth referred to as the “Afro-Arabian” region) harbours a unique mix of fauna and flora shaped by the harsh climatic conditions and the long-term geographical connectivity between Africa and Asia^20^. Therefore, many of the Afro-Arabian species are endemic and uniquely adapted to extreme environmental conditions^2,8,19,21^.

Climatic conditions limit mammal distribution by influencing physiological processes and vegetation conditions, which in turn determine resource availability^22^. The mammal fauna of the Afro-Arabian region, and in particular the endemic species, are among the most adapted to the extreme and harsh environmental conditions^8,23^. However, evidence suggests that the genetic adaptation in mammals can not keep pace with the current rate of climate change^24^ making living at the edge of the physiological limits particularly risky^24,25^. Mammals, as consumers, are at higher trophic levels, and have a unique functional role in the ecosystem^2^; therefore, focusing our attention on them could be a useful surrogate for greater biodiversity conservation and a suitable way to prioritise the conservation effort^22^.

The long history of conflict, political instability, and perception as a bare area with comparably low biomass, are responsible for the lack of information regarding the biodiversity patterns and species distributions in the Afro-Arabian region^2,8,26^. In addition, despite their important functional roles in the ecosystem, these species have also been understudied because of the difficulties associated with studying the often nocturnal, rare and secretive species^1,2^. Such lack of information represents the main challenge for any conservation action.

The main goal of this study, which also forms part of a published PhD thesis^27^, is to identify the conservation status and diversity patterns of the endemic mammal species in the Afro-Arabian region based on species distribution models (SDMs). Species distribution models allow for delineating species geographic and environmental niches through quantifying the relationships between their occurrences and associated biotic and abiotic variables, and allow for predicting species niches in unsampled regions^28,29^. SDMs are powerful tools and widely used in many fields (e.g., biogeography, ecology, conservation, and epidemiology) and can project species niche spatially and temporally^30^. Specifically, we aim to address the following questions:

- Where are the biodiversity hotspot areas located?
- What is the expected impact of climate change on biodiversity in the Afro-Arabian region?
- What would the conservation status of focal species be under climate change?

We delineated the contemporary potential distribution ranges for the endemic mammal species of the Afro-Arabian region using SDMs. Then, based on the range maps derived from the SDMs, we defined the biodiversity hotspot areas in the study area. We then projected the current potential ranges temporally into 2050 and 2070 to predict the potential impact of climate change on the distribution of the endemic mammals in the future. According to the degree of predicted change in the species’ ranges, we then assigned a threat category to each species following the IUCN Red List criterion A3(c).

## Results

**Figure 2:**
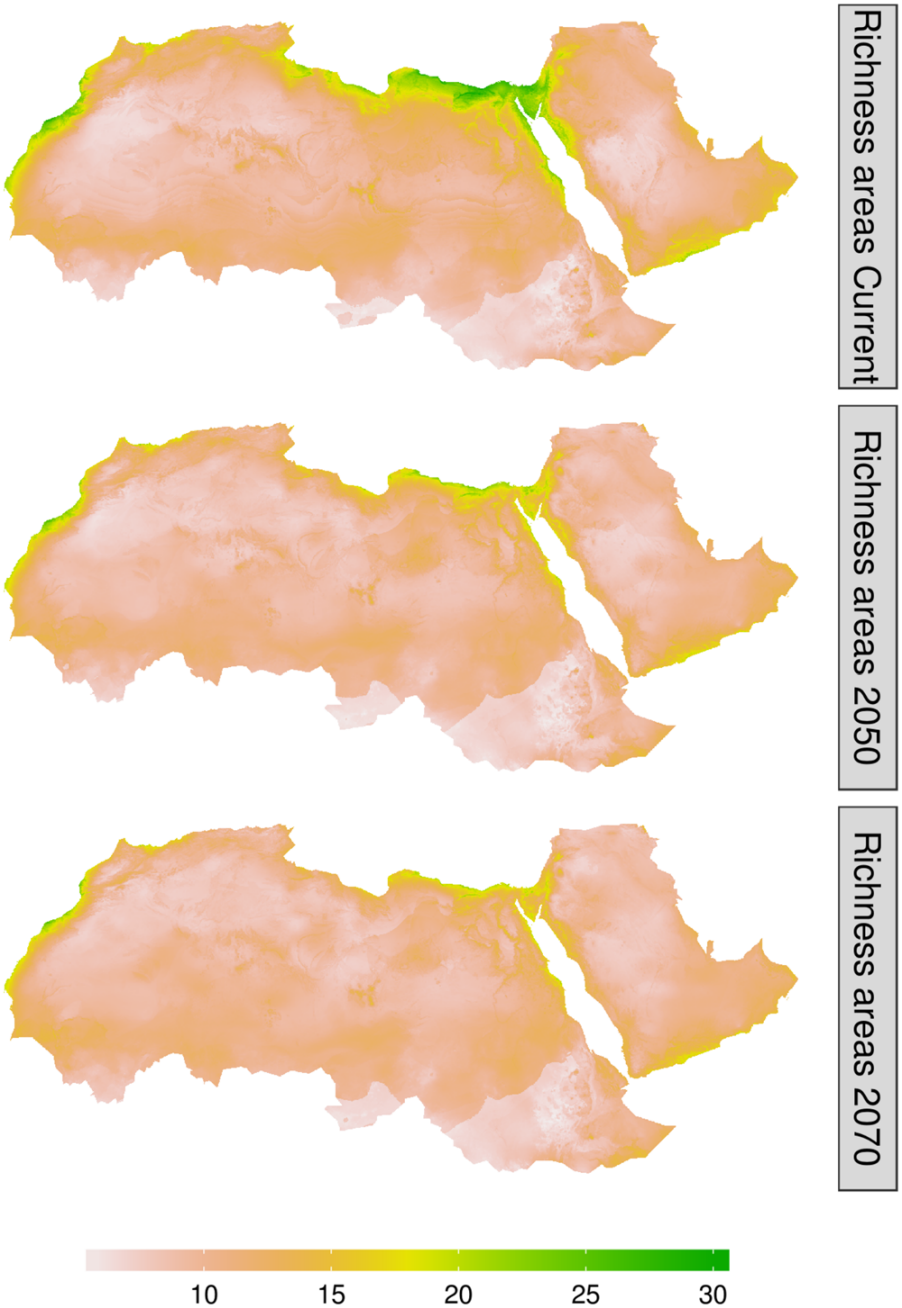
Species richness areas of the endemic mammal species in the Afro-Arabian region along three time slices. The “Current” refers to the contemporary status of the species richness, 2050 and 2070 refer to the predicted status in those years. The scale bar shows the score of the species richness, where zero means almost no species and 30 means the number of species in the pixel.

We compiled data for 107 endemic mammal species belonging to 17 families (Supplementary Table S1). The most represented families were Muridae (41 species), Soricidae (18 species), and Bovidae (16 species). All ensemble SDMs had a good predictive performance (True Skill Statistic (TSS) > 0.49, mean = 0.80, SD = 0.09, and Area Under the Receiver Operator Curve (AUC) > 0.74, mean = 0.89, SD = 0.05) suggesting that the SDMs were well capable to identify suitable areas within the study region (AUC & TSS values for each species are listed in Supplementary Table S2). The spatial analysis revealed that the contemporary species richness reaches a maximum of 35 endemic mammal species per ~25 km^2^. The areas with the highest richness values were concentrated along the coast of the Mediterranean and Red sea, and around the mountainous regions. The areas with the lowest richness were found in central Algeria and Mauritania in the West and central Saudi Arabia in the East (Fig. 2). It is noteworthy that the species richness showed a latitudinal gradient for endemic mammal species increasing northward.

**Figure 3:**
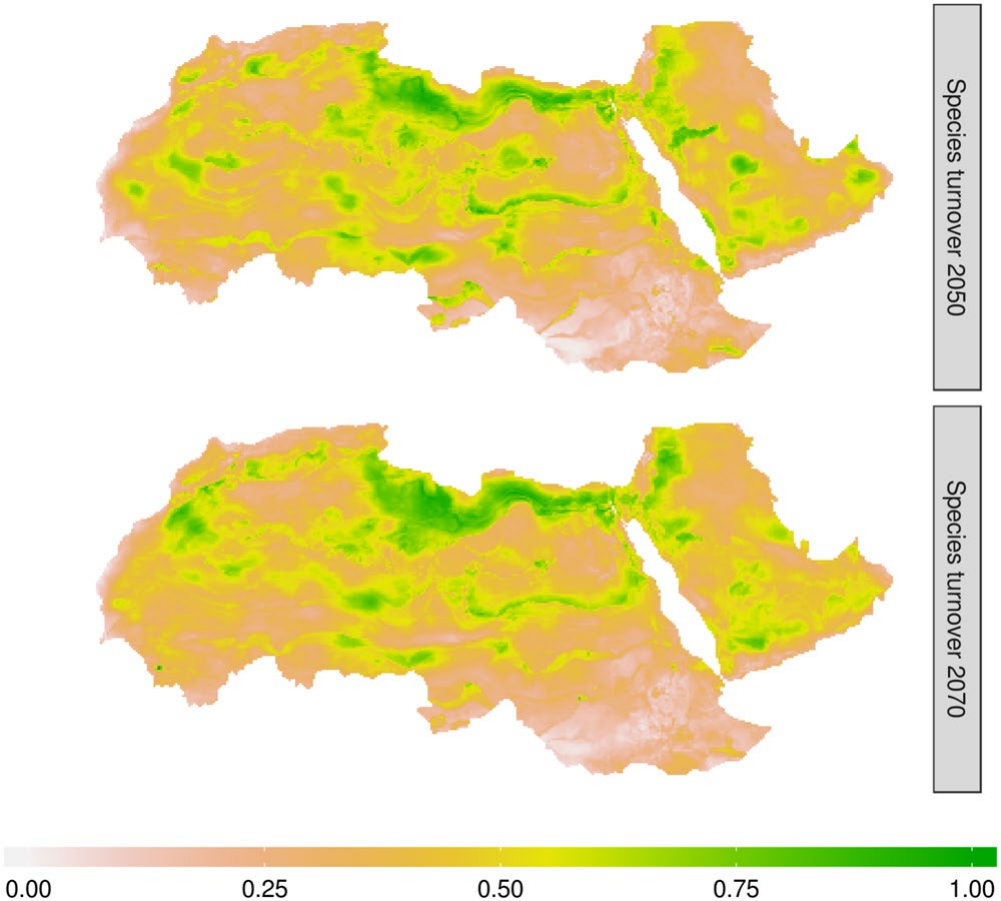
Temporal species turnover of the endemic mammal species in the Afro-Arabian region. The zero value indicates no change in species composition over time (2050 and 2070) and the value of one indicates a complete change in species composition.

Our results also revealed that both species richness and turnover showed significant temporal trends, where the projected change in climate conditions resulted in markedly reduced mammalian diversity in the study area. We found, under the Unlimited Dispersal scenario (UD) assuming that the species will be able to disperse freely to the projected suitable habitats, about 17% reduction in the species richness by 2050, increasing to 20% by 2070. Similarly, species turnover—the change in species composition over time— was predicted to increase steadily during the 21st century (Fig. 3). The changes in species turnover were predicted to be higher particularly in the central Sahara and the western side of the Arabian Desert.

**Figure 4:**
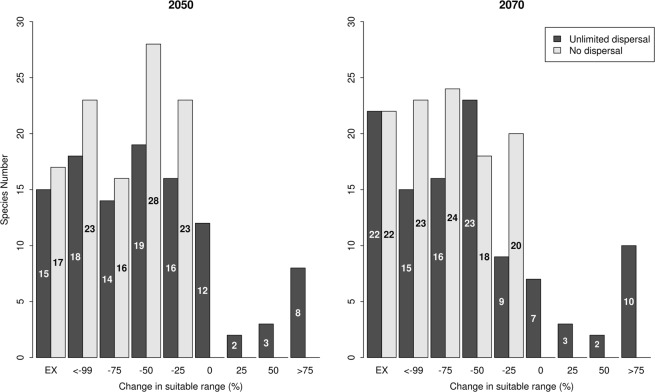
The percentage of change in climatically-suitable habitat for the endemic mammal species in 2050 (left) and 2070 (right) inhabiting the Afro-Arabian region. The negative numbers on the x-axis indicate loss in suitable habitat. The numbers on the bars represent the number of species in each category.

The impact of the projected climate change scenarios on the potential species distribution was not consistent and varied among the species, where some were predicted to reduce the size of their distribution ranges and become threatened, while other species were predicted to expand their distribution ranges and thus benefit from climate change. According to the No Dispersal scenario (ND), assuming that the species will not be able to occupy the projected suitable habitats, 17 and 22 species were predicted to go extinct (losing 100% of their suitable range) by 2050 and 2070 respectively. Under the same scenario, 84 and 87 species were predicted to lose more than 30% of their suitable ranges by 2050 and 2070 respectively (Fig. 4 and Supplementary Table S3). In the unlimited dispersal scenario, the models predicted 15 and 22 species to go extinct by 2050 and 2070 respectively, while 15 species were predicted to expand their distribution ranges over that time (Fig. 4). The majority of the species at risk of going extinct were small mammals belonging to the family Soricidae (seven out of 21) and the family Muridae (ten out of 42). In general, about 80 endemic mammal species in the Afro-Arabian region will be likely to become severely threatened (losing 50–100% of their current potential suitable habitats) according to the projected climate change (Fig. 4).

**Figure 5:**
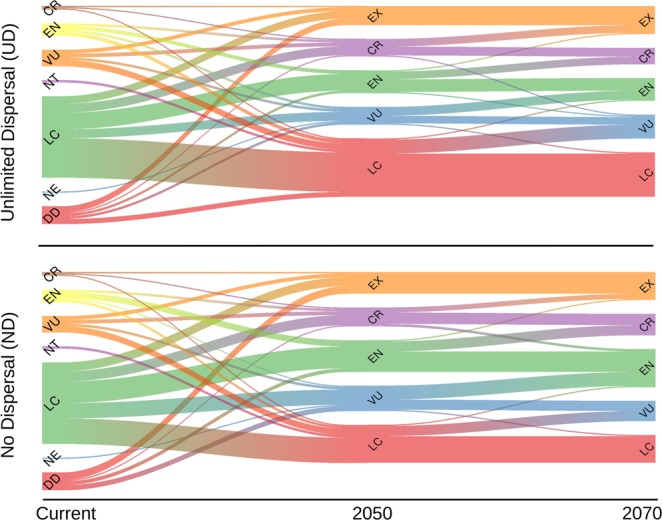
Changes in the threat category for the endemic mammal species in the Afro-Arabian region over time. The upper plot represents the unlimited dispersal scenario (UD), while the lower plot represents the no dispersal scenario (ND). The x-axis represents the temporal scale, where the current refers to the current species conservation status according to the IUCN, while 2050 and 2070 are the predicted conservation status according to the change in distribution range in those respective years.

Our results show a striking difference between the current IUCN conservation status and the predicted status according to the projected change in the distribution range (Fig. 5). About 50% of the species currently classified as “Least Concern” are expected to be classified in the near future anywhere between the categories “Vulnerable” to even “Extinct” (according to the Unlimited Dispersal scenario).

## Discussion

Based on the best available species distribution models, we examined how the projected climate change could influence the distribution and diversity patterns of endemic mammals in the Afro-Arabian region. Our results show that many of the endemic mammals are facing an extremely augmented risk of extinction in the immediate future (Fig. 4 and Supplementary Table S3). This result supports the findings of previous studies that have shown that a substantial number of vertebrate species globally are expected to become extinct in the next decade^3,31^. This high extinction risk could be amplified by the influence of additional drivers such as changing land-use patterns, disease outbreaks, human pressure, and introduction of novel parasites or invasive species^32,33^. In fact, the impact of these drivers is already obvious in the Afro-Arabian region, in particular catalysed by the absence of law enforcement^32^. Additionally, changing rainfall patterns due to climate change can increase a species’ susceptibility to disease outbreaks, exemplified by the population decline in the Kruger park as a result of an Anthrax outbreak after the 1990 drough^22^.

In contrast, species ecological traits could also dampen the impact of climate change. Some species may show enough phenotypic or behavioural plasticity to adapt to new climate condition^34^. For instance, studies showed that the bushy-tailed woodrat, *Neotoma cinerea*, increased in body mass to adapt to low temperatures in the Late Quaternary, and decreased it to adapt to the recent increases in temperature^34–36^. For some species, particularly small mammals, avoiding high temperatures can be achieved by finding shelter such as burrows^22^. Other species can avoid the effects of rising temperature by employing “adaptive heterothermy” a physiological mechanism to reduce heat gain and evaporative heat loss and thus conserving body water^25,37^. Accordingly, if species succeed to adapt to local conditions, the impact of climate change may not be as pronounced as projected^22^.

For those species predicted to expand their climatically suitable ranges (Fig. 4), it will be important to have a dispersal rate that conforms with the climatic velocity (i.e. the rate at which these species disperse should keep pace with the change in climate)^38–40^. In the Afro-Arabian region, which is dominated by high climatic velocity, the species dispersal ability is either naturally low or artificially limited by habitat fragmentation. Furthermore, biotic interactions and the existence of natural and anthropogenic barriers further limit the species ability to expand or shift their distribution ranges^5,38,39^. Assuming that species would be able to disperse to the new climatically-suitable habitats, the species still may not be able to colonise the new habitats if it is not meeting the requirements^34^. The ability of these species to migrate, especially herbivorous mammals, is limited by the mobility of the plant species required as food^34^. Whereas particularly the predators are intimately limited by prey availability rather than just dispersal capacities to the new generally climatically-suitable habitats^22^.

Our results show a high divergence between the current IUCN conservation status and the predicted status according to the projected change in the distribution range (Fig. 5). This disagreement is owed to applying different criteria to assess species extinction. For instance, our assessment is based on the A3(c) criterion that assign species to a threat category according to the degree of change in species’ ranges. Other criteria use other factors (e.g., the size of distribution range, population status, number of sub-population, and threats severity) to assign species to a threat category. As such, species can be considered threatened according to a specific criterion and not threatened according to another. We acknowledge that SDMs do not identify the proximate causes of species extinction^12^, however, the reduction in species distribution range is very likely to increase the risk of local extension^41^. For instance, species predicted to be or become range restricted to a few locations, could easily go extinct by any further local threat such as land transformation or disease outbreaks^12^. In any case, our findings, together with the results of previous studies, advocate for considering the impact of projected climate change in future assessments calling for immediate action^12,22,34,42^.

The latitudinal gradient in the current species richness corroborates the pattern for other taxa^43–46^. This pattern could be attributed to factors such as palaeogeography and/or historical land-use patterns which complemented each other to form a mosaic of interconnected habitats, resulting in high species richness in the northern parts of the study area^47,48^. Additionally, areas with high species richness are also geographic transition zones. For instance, the Sinai is a transitional habitat between Asia and Africa, while Morocco lies between Africa and Europe. These transition habitats are characterised by high levels of species diversity and endemism, and the occurrence of species with allopatric ranges^49^.

Although species richness was predicted to decrease and hotspot areas to become isolated during the next decades, the regions with high richness were predicted to remain around the coastal areas and in the Sinai region (Fig. 3). Previous studies also revealed the importance of these regions for many other taxa at global and local scales^32,44,45,50–54^. However, the political conflicts, particularly in the Sinai region, and the sprawling development along the coastal areas do not hold much promise for the survival of species in these regions. An accurate assessment of biodiversity and its threats is not available across the Afro-Arabian region because of its remoteness and political instability. However, the rate of hunting wildlife unsustainably substantially increased and several wildlife massacres have been reported particularly after the “Arab spring” in 2011^26,55,56^. Recent analyses show that the increased conflict and anthropogenic activities resulted in a dramatic population decline for many species^55,57,58^. Additionally, most of the megafauna in the southern part of the study area have been almost extinguished because of armed conflicts^55^. The current conflicts and instability together with other disturbances (mining, grazing, agriculture, and urbanisation), leave endemic species with nowhere to go, amplifying the risk of extinction to be realistically much more dramatic than our projections^2,55,58^.

It is noteworthy that the regions with high species turnover corresponded to the regions with low species richness, except in the Sinai region (Fig. 3). This could be because the species assemblages in our study follow the hollow curve pattern, where most species have small distribution ranges with just a few large ones^59–61^. The change in species composition reflected by high species turnover, due to species range contractions and expansions, as well as influx of new species and/or departure of other species, could negatively influence the existing species interaction patterns and the trophic network structure^22,34,42^.

Our results should be interpreted cautiously, given some of the inherent uncertainties associated with SDMs being projected into the future. A primary source of uncertainty in our study is the quality and quantity of species occurrences. In this study, the distribution ranges for about 20% of the focal species were modelled with low quantities of five to 10 occurrences. Using such small numbers for modelling is challenging and likely to reduce prediction accuracy. However, we minimised model uncertainty by avoiding model complexity using fewer predictors (i.e. equal to or less than the number of occurrences) which should reduce the risk of model over-fitting^62^. Additionally, we used SDM algorithms that proved to have good performance with low numbers of occurrences^63^. Since our data was collected from different sources with different sampling approaches, it certainly contained sampling bias, which often results in environmental bias (i.e. over‐representation of certain environments) and, in turn, results in truncated response curves and ultimately yields less reliable predictions^64,65^. We used, however, a spatial filtering approach to address such potential bias in the species occurrence data. Another sources of uncertainty comes from model extrapolation, projecting models onto non-analogue environments that were not used for calibration^62,66^. To minimise model extrapolation bias and improve the prediction reliability, we projected the current distributions onto the future climate scenarios using the ‘clamping’ option, which allows some extrapolation onto non-analogue environments, but in a constrained manner. We acknowledge that there is also another more conservative approach, which completely avoids model extrapolation, however, such an approach can produce truncated response curves and even less reliable projections^67^. We are not claiming that our projections have no extrapolation bias (they certainly have), however, this extrapolation bias is restricted and reasonable from a practical stand point. A further source of uncertainty is variable selection and availability. Information, for example, on how vegetation variables (VCF) might change in the future is not available, accordingly we assumed it to remain (i.e. static variable) unchanged. It is therefore crucial to understand that our projections are only valid under the assumption we had to make. However, since most of the species with distribution ranges shaped by VCF were bats, which have the ability to disperse and track changes in VCF that could happen in the future, we do not expect that changing VCF in the future will significantly change the conclusions of our study.

## Conservation Implication

For systematic conservation planning and setting conservation priorities it is imperative to know about species distributions and their relationship with biotic and abiotic factors^68–70^. However, despite their ecological roles in the ecosystem, particularity in the Sahara and the Arabian Desert, mammal species are widely neglected from conservation plans, and not even fully recognised by the local governments^2,19,27^. The ecological role that many mammal species play makes them ideal umbrella or keystone species. Prioritising the conservation efforts towards these species would provide collateral protection to co-occurring species^22^. For instance, the recruitment of *Acacia* species, a keystone species in the Sahara and the Arabian Desert, increased with the presence of herbivore species (e.g. Dorcas gazelle *Gazella dorcas*)^71^. Unlike other herbivorous species, Dorcas gazelles are known to be browsers and not grazers, which, in turn, allows the vegetation cover for the desert ecosystem to survive and persist, in addition they also act as plant seeds dispersers^72–74^. At the same time, the gazelles are the main prey for several predators (e.g. Cheetah *Acinonyx jubatus* and Wolf *Canis lupus*) that have suffered from decline in prey availability^75^.

Our results, together with other studies^5,10,26^ all show that biodiversity in the Afro-Arabian region is highly vulnerable to projected climate change. To save what is left, conservation measures should prioritise the hotspot areas, i.e. regions with a high number of endemic species. Vanishing species and populations will potentially affect the existing ecological network as some species may experience ecological release and others may be suppressed causing cascades of extinctions^42,76^. The Afro-Arabian species exhibit high physiological plasticity and distinct evolutionary histories that provide valuable insights into the genetic basis of species tolerance to environmental extremes^2,4,5^. Thus, losing these endemic species could lead to a loss in the pool of important genetic information that is vital for understanding species’ evolution and survival in extreme environments^4^. As such, conservation efforts need to maximise species dispersal within their suitable habitat; this would slow down the rate of extinction by maintaining the interaction between species and populations and preserving the genetic network^77^. We highly recommend applying adaptive conservation strategies inside and outside protected areas as a first critical step forward^32,78^. These strategies include three options: 1) the *resistance* option, reducing the threat impacts and conserving valued resources (e.g., reducing non-climatic stress—invasive species and habitats fragmentation— and promoting green energy), 2) the *resilience* option, restoring the ecosystem functions (e.g., improving protected area networks and habitats connectivity), and 3) the *response* option, allowing the ecosystem transition to new conditions (e.g., using transboundary protected areas)^32,78^. The existing protected area networks can be used to initiate the adaptive conservation strategies. Increasing redundancy in the existing protected area networks can provide species with more opportunities to move and disperse, which, ultimately, improves species resilience to climate change^79^. The existing protected area networks in the Afro-Arabian region are few, small in size, and spatially disconnected^32^. Therefore, expanding the protected area networks is likely to preserve various environmental conditions, improve habitat connectivity, and facilitate species dispersal along different environmental gradients^77^. Additionally, we recommend establishing new protected areas to cover the regions predicted to experience high turnover rates (Fig. 3), and managing the surrounding habitats in ways that allow for species dispersal and movement. The socio-economic aspect and community-based conservation approach need to be considered in the conservation management plan to achieve a long-term conservation success^32^.

## Methods

### Study area

This study focuses on the Afro-Arabian region which covers ~17,820,000 km^2^, and extends over 26 countries in Africa and Asia (Fig. 1). It covers two deserts, the Saharan and the Arabian Desert, that occupy the southern part of the western Palearctic and extend also into the Afro-tropical realm^27^. The region is bordered by the Mediterranean Sea in the north, Persian Gulf in the east, the Atlantic Ocean in the west, and the political borders of the Sahel countries in the south (Fig. 1). This region has relatively high topographic heterogeneity (altitude varies from ~−400 to 4000 meters a.s.l.) and habitat diversity (sand dunes, coastal plains, mountains). It encompasses mainly three distinct climatic regions: warm Mediterranean, warm semi-arid, and warm desert^80^. The latter climate region encompasses the Sahara biome, the largest warm desert in the world, with mean annual temperature of ~32 °C and mean annual precipitation of zero^26^, and spreading over ~80% of the study region. The study region experienced environmental and geological oscillations in the past that allowed for faunal interchange between Africa and Asia^20^.

### Species data

Point occurrences for 107 mammal species (Supplementary Table S1), specifically 95 endemic and 12 near-endemic species (>70% of their distribution ranges within the study region), were assembled from different resources, including personal fieldwork, published and “gray” literature, online portals, and personal communications (Supplementary Table S4). We updated species taxonomy following the Mammal Species of the World portal^81^. We restricted the species records to those only collected from the wild between 1960 and 2005, and omitted the duplicated occurrences within one grid cell (~5 × 5 km). To correct for sampling bias, we applied a spatial filtering approach^82,83^. Specifically, we filtered records of each species to obtain the highest achievable occurrences that are 5, 10, and 20 km apart for small, volant, and large mammals respectively. We chose these distances to represent the mean dispersal ability of each group. This should lead to less spatially autocorrelated records with less environmental bias, and yield better prediction for species distribution ranges^84^. We acknowledge that there are several bias correction approaches such as calibrating the model with extra predictors representing site accessibility or sampling effort, or background manipulation^85,86^. However, in this study we favoured spatial filtering since it has been shown to outperform other bias correcting approaches^87–90^. As a consequence, it has been widely applied recently to correct the spatial and environmental bias in species data^83,90–94^. Spatial filtering outweighs the negative influence of the biased data and substantially minimises omission errors (i.e. suitable areas not predicted by models) and commission errors (i.e. areas mistakenly predicted to be suitable) particularly in areas with limited surveying effort^87^. (For assessing the environmental bias see Supplementary Methods “Environmental sampling bias assessment” & Supplementary Figure S1).

### Environmental data

We initially selected 26 environmental predictors, 19 contemporary climatic predictors available from the WorldClim database and seven non-climatic predictors characterising the topography and vegetation, at a resolution of 2.5 arc-min (Supplementary Table S5), to model species distributions. This resolution allows for mitigating the uncertainty that might result from the positional uncertainty associated with the species occurrences, and also for avoiding the issue of not identifying local refugia, a common issue in coarse spatial resolution climate change projections found on correlative SDMs^32,95^. Model over-fitting is a problem in the presence of multi-collinearity between the predictors^96,97^. We therefore calculated the collinearity between the predictors based on the Variance Inflation Factor (VIF), and removed the predictors with VIF values greater than 10^98^, using the “vifstep” function in “usdm” R package^99^. This reduced the number of predictors to 13 (Supplementary Tables S5 & S6). To assess the potential impact of climate change on the distribution of the focal species, we retrieved projections of future climate variables from four general circulation models (GCMs), HadGEM2-AO, CCSM4, IPSL-CM5A-LR, and ACCESS1-0, downscaled to 2.5 arc-min resolution from the IPCC Fifth Assessment, available from the WorldClim database for the years 2050 and 2070^100^. We used the VIF to assess collinearity within future predictors (2050 and 2070) and to avoid making predictions based on collinearity structures in time^101^ (Supplementary Table S6).

We used the RCP 8.5 pessimistic emission scenario that assumes increasing in the global population and slow per capita economic growth, leading to high energy demand and high GHG emissions and the average temperature over time in absence of climate change policies^102,103^. The Afro-Arabian region, in many of its countries, is suffering from civil conflicts and wars, political unrest, increasing population size, low per capita income, and absence of climate change policies^55,56^. Therefore, we restricted our analysis to the RCP 8.5 scenario as most realistic scenario (or even the most pessimistic), for the Afro-Arabian region, that is probably representing the actual situation in the study area and also bears only the risk of our results being a conservative outcome. Finally, since there is no data on the ‘vegetation continuous field’ (VCF) variable^104^ and how it might change in the future, we assumed that VCF remains constant to be able to include it in the future predictions^105,106^. To avoid prediction unreliability owing to non-analogue environments in the future (2050 and 2070), we used ExDet tools^107^ to identify the presence of non-analogue environments and the extent of model extrapolation (see Supplementary Methods “non-analogue environments and extrapolation” & Supplementary Figures S2 and S3).

### Species Distribution Models (SDMs)

We adopted ensemble SDMs with the weighted average consensus approach that accounts for model-based uncertainty^108,109^ to delineate the contemporary and future distribution ranges. The ensembles consisted of four different SDM algorithms: MaxEnt^110^, Random Forest (RF)^111^, Generalized Boosted Model (GBM)^112^, and Generalized Linear Model (GLM)^113^, available within the “biomod2” R package^114^. However, for each species, the selection of SDM algorithms was based on the number of species occurrences and species specialisation following the framework developed by Soultan and Safi^27,63^. To minimise the model complexity and avoid model over-fitting^82,83^, we calibrated an initial model for each species using the filtered species occurrences and the uncorrelated current environmental predictors. Subsequently, for each species, we calculated the variable importance of each predictor to model gain, retaining those with a relative contribution greater than 5% for the final model calibration (Supplementary Table S7)^115–117^. To avoid geographical over-prediction, we used a species-specific study area^118^. Specifically, we restricted the model calibration to the buffered minimum convex polygon (MCP) of the species occurrences, where the buffer distance was equal to the mean distance between the occurrence points of the focal species^83,84,119^. This was done to avoid model over-fitting when the environmental conditions in species occurrences were different from those in background locations^120–122^. We thus randomly sampled 10’000 background points, constrained to within the buffered area, to characterise the environmental background^122^.

Finally, we projected the model predictions onto the Afro-Arabian region to determine the contemporary potential distribution for each species. For species with a large MCP (>50% of the study area), we used the entire study area for model calibration. We used spatial cross-validation to avoid calibrating and evaluating based on the same data^50,83^. To this end, we segregated species localities geographically into four blocks, then, models were built in an iterative manner, using three blocks for calibration and the remaining fourth block for evaluation in each iteration, until all blocks have been used once for evaluation^123,124^. This allowed for: 1) maintaining spatial independence between training and test data, 2) avoiding AUC inflation due to spatial auto-correlation between training and test data and, 3) incorporating the uncertainty in the estimated probability^123,124^. We evaluated the predictive performance of the models by assessing their ability to predict relative likelihoods of species occurrence within the test data blocks, using the area under the curve (AUC) of the receiver operating characteristic (ROC) plot^125^, as well as the True Skill Statistic (TSS)^126^. We projected the contemporary species distribution ranges into the future (2050 and 2070) in order to assess the possible consequences of climate changes on the biodiversity in the Afro-Arabian region. There is large variability among general circulation models (GCMs), which can cause uncertainties in species distribution projections^123,127–129^. Accordingly, to reduce the bias in certain areas from one GCM, we calculated the median of the predictions from the four GCMs^129–132^. This approach showed to provide accurate and less biased predictions and provides better consideration for the uncertainties in future species distribution^129,131,133^.

### Spatial analysis

We quantified the impact of global change on the spatial biodiversity patterns through two indices: species richness and species turnover. Species richness is important for understanding the ecological and evolutionary processes that shape the biodiversity in a given location and is one of essential measures for prioritising conservation efforts^8,134^. We estimated species richness for current and future, 2050 and 2070, by summing up the predicted relative likelihoods of species occurrence for all species for the corresponding time^34,124^. Species turnover is a measure of dissimilarity in the composition of species assemblages from site to site within a given time frame or over time within a given site^135^. We used the Bray-Curtis index to quantify the “temporal” species turnover, dissimilarity for each pixel between the current and future, using the “vegdist” function in the “vegan” R package^136^.

Additionally, we accounted for the ability of species to track the changes in climate using two dispersal scenarios: Unlimited Dispersal (UD), assuming that the species can disperse freely and adjust their distribution range according to climate, and No Dispersal (ND), where species are assumed to be unable to disperse and, as a consequence, will not be able to occupy the projected suitable habitats. According to the rate of change in species distribution ranges, we assigned each species to a threat category following the International Union for Conservation of Nature (IUCN) threat categories^137^. We applied the following criteria as described in IUCN Red List criterion A3(c): Extinct when a species is predicted to lose 100% of its current potential range; Critically Endangered when a projected range loss is >80%; Endangered projects a range loss of >50%; and Vulnerable when a projected range loss is >30%^9,12,34^. IUCN Red List criterion A3(c) estimate the rate of geographic range decline over a period of 10 year or three generations, whichever is the longer^138^. Since 10 years is a short period to measure the impact of climate change and also does not reflect time scales for human interventions^138^, we used the generation definition and considered each time period (2050 and 2070) as equal to three generations.

## Supplementary information

Supplementary information

## References

[1] Levin PS, Levin DA (2002). Macroscope: The Real Biodiversity Crisis. Am. Sci..

[2] Durant SM (2014). Fiddling in biodiversity hotspots while deserts burn? Collapse of the Sahara’s megafauna. Divers. Distrib..

[3] WWF. (2016). Living Planet Report 2016. Risk and resilience in a new era.

[4] Ceballos G, Ehrlich PR, Dirzo R (2017). Biological annihilation via the ongoing sixth mass extinction signaled by vertebrate population losses and declines. Proc. Natl. Acad. Sci. USA.

[5] Vale CG, Brito JC (2015). Desert-adapted species are vulnerable to climate change: Insights from the warmest region on Earth. Glob. Ecol. Conserv..

[6] CBD. *Strategic Plan for Biodiversity 2011*–*2020*. *Further information related to the technical rationale for the Aichi Biodiversity Targets*, *including potential indicators and milestones*. *UNEP*/*CBD*/*COP*/*10*/*INF*/*12*/*Rev*.*1*. *Convention on Biological Diversity* (2010).

[7] Visconti P (2016). Projecting Global Biodiversity Indicators under Future Development Scenarios. Conserv. Lett..

[8] Vale CG (2016). Biogeography and conservation of mammals from the West Sahara-Sahel: an application of ecological niche-based models and GIS. Hystrix, Ital. J. Mammal..

[9] Thuiller W (2011). Consequences of climate change on the tree of life in Europe. Nature.

[10] Sow AS, Martínez-Freiría F, Dieng H, Fahd S, Brito JC (2014). Biogeographical analysis of the Atlantic Sahara reptiles: Environmental correlates of species distribution and vulnerability to climate change. J. Arid Environ..

[11] Gibson L, McNeill A, Tores Pde, Wayne A, Yates C (2010). Will future climate change threaten a range restricted endemic species, the quokka (*Setonix brachyurus*), in south west Australia?. Biol. Conserv..

[12] Thuiller W, Lavorel S, Araújo MB, Sykes MT, Prentice IC (2005). Climate change threats to plant diversity in Europe. Proc. Natl. Acad. Sci. USA.

[13] Rondinini C (2011). Global habitat suitability models of terrestrial mammals. Philos. Trans. R. Soc. London B Biol. Sci..

[14] Boitani L (2008). Distribution of medium- to large-sized African mammals based on habitat suitability models. Biodivers. Conserv..

[15] Record S, Charney ND, Zakaria RM, Ellison AM (2013). Projecting global mangrove species and community distributions under climate change. Ecosphere.

[16] Ceballos G, Brown JH (1995). Global Patterns of Mammalian Diversity, Endemism, and Endangerment. Conserv. Biol..

[17] Christianini AV, Oliveira PS, Bruna EM, Vasconcelos HL (2014). Fauna in decline: Meek shall inherit. Science..

[18] Moura MR, Argôlo AJ, Costa HC (2017). Historical and contemporary correlates of snake biogeographical subregions in the Atlantic Forest hotspot. J. Biogeogr..

[19] Durant SM (2012). Forgotten Biodiversity in Desert Ecosystems. Science..

[20] Metallinou M (2012). Conquering the Sahara and Arabian deserts: systematics and biogeography of Stenodactylus geckos (Reptilia: Gekkonidae). BMC Evol. Biol..

[21] Brito JC (2016). Conservation Biogeography of the Sahara-Sahel: additional protected areas are needed to secure unique biodiversity. Divers. Distrib..

[22] Thuiller W (2006). Vulnerability of African mammals to anthropogenic climate change under conservative land transformation assumptions. Glob. Chang. Biol..

[23] Ward, D. Biodiversity and Biogeography of Deserts. In *The Biology of Deserts* 192–216, 10.1093/acprof:oso/9780198732754.003.0009 (Oxford University Press, 2009).

[24] Hetem RS, Fuller A, Maloney SK, Mitchell D (2014). Responses of large mammals to climate change. Temp. (Austin, Tex.).

[25] Hetem RS (2010). Variation in the daily rhythm of body temperature of free-living Arabian oryx (*Oryx leucoryx*): does water limitation drive heterothermy?. J. Comp. Physiol. B.

[26] Brito JC (2014). Unravelling biodiversity, evolution and threats to conservation in the Sahara-Sahel. Biol. Rev. Camb. Philos. Soc..

[27] Soultan, A. *Biogeography and conservation of the neglected biodiversity*. (University of Konstanz, 2018).

[28] Carvalho SB, Brito JC, Pressey RL, Crespo E, Possingham HP (2010). Simulating the effects of using different types of species distribution data in reserve selection. Biol. Conserv..

[29] Guisan A (2013). Predicting species distributions for conservation decisions. Ecol. Lett..

[30] Elith J, Leathwick JR (2009). Species Distribution Models: Ecological Explanation and Prediction Across Space and Time. Annu. Rev. Ecol. Evol. Syst..

[31] Ripple WJ (2015). Collapse of the world’s largest herbivores. Sci. Adv..

[32] Maiorano L (2011). The future of terrestrial mammals in the Mediterranean basin under climate change. Philos. Trans. R. Soc. B Biol. Sci..

[33] Hoffmann M (2011). The changing fates of the world’s mammals. Philos. Trans. R. Soc. B Biol. Sci..

[34] Levinsky I, Skov F, Svenning J-C, Rahbek C (2007). Potential impacts of climate change on the distributions and diversity patterns of European mammals. Biodivers. Conserv..

[35] Smith FA, Browning H, Shepherd UL (1998). The influence of climate change on the body mass of woodrats Neotoma in an arid region of New Mexico, USA. Ecography..

[36] Smith FA, Betancourt JL (1998). Response of Bushy-Tailed Woodrats (*Neotoma cinerea*) to Late Quaternary Climatic Change in the Colorado Plateau. Quat. Res..

[37] Schmidt-Nielsen K, Schmidt-Nielsen B, Jarnum SA, Houpt TR (1956). Body Temperature of the Camel and Its Relation to Water Economy. Am. J. Physiol. Content.

[38] Loarie SR (2009). The velocity of climate change. Nature.

[39] Carroll C (2015). Biotic and Climatic Velocity Identify Contrasting Areas of Vulnerability to Climate Change. PLoS One.

[40] Menéndez R, González-Megías A, Lewis OT, Shaw MR, Thomas CD (2008). Escape from natural enemies during climate-driven range expansion: a case study. Ecol. Entomol..

[41] Thomas CD (2004). Extinction risk from climate change. Nature.

[42] Velásquez-Tibatá J, Salaman P, Graham CH (2013). Effects of climate change on species distribution, community structure, and conservation of birds in protected areas in Colombia. Reg. Environ. Chang..

[43] Patiny S, Michez D (2007). Biogeography of bees (Hymenoptera, Apoidea) in Sahara and the Arabian deserts. Insect Syst. Evol..

[44] Patiny S, Michez D, Kuhlmann M, Pauly A, Barbier Y (2009). Factors limiting the species richness of bees in Saharan Africa. Bull. Entomol. Res..

[45] Bilgin R, Keşişoğlu A, Rebelo H (2012). Distribution Patterns of Bats in the Eastern Mediterranean Region Through a Climate Change Perspective. Acta Chiropterologica.

[46] Myers N, Mittermeier RA, Mittermeier CG, Da Fonseca GAB, Kent J (2000). Biodiversity hotspots for conservation priorities. Nature.

[47] Medail F, Quezel P (1999). Biodiversity Hotspots in the Mediterranean Basin: Setting Global Conservation Priorities. Conserv. Biol..

[48] Covas R, Blondel J (2008). Biogeography and history of the Mediterranean bird fauna. Ibis (Lond. 1859)..

[49] Bilgin R, Karataş A, Çoraman E, Disotell T, Morales J (2008). Regionally and climatically restricted patterns of distribution of genetic diversity in a migratory bat species, *Miniopterus schreibersii* (Chiroptera: Vespertilionidae). BMC Evol. Biol..

[50] Tisseuil C, Leprieur F, Grenouillet G, Vrac M, Lek S (2012). Projected impacts of climate change on spatio-temporal patterns of freshwater fish beta diversity: a deconstructing approach. Glob. Ecol. Biogeogr..

[51] García Molinos J (2015). Climate velocity and the future global redistribution of marine biodiversity. Nat. Clim. Chang..

[52] Ficetola GF, Bonardi A, Sindaco R, Padoa-Schioppa E (2013). Estimating patterns of reptile biodiversity in remote regions. J. Biogeogr..

[53] Jones MC, Cheung WWL (2015). Multi-model ensemble projections of climate change effects on global marine biodiversity. ICES J. Mar. Sci..

[54] Leach K, Zalat S, Gilbert F (2013). Egypt’s Protected Area network under future climate change. Biol. Conserv..

[55] Brito, J. C. *et al*. Armed conflicts and wildlife decline: Challenges and recommendations for effective conservation policy in the Sahara-Sahel. *Conserv*. *Lett*. e12446, 10.1111/conl.12446 (2018).

[56] Daskin, J. H. & Pringle, R. M. Warfare and wildlife declines in Africa’s protected areas. *Nature*, 10.1038/nature25194 (2018). 10.1038/nature25194 29320475

[57] Duncan C, Kretz D, Wegmann M, Rabeil T, Pettorelli N (2014). Oil in the Sahara: mapping anthropogenic threats to Saharan biodiversity from space. Philos. Trans. R. Soc. Lond. B. Biol. Sci..

[58] Newby, J., Wacher, T., Durant, S. M., Pettorelli, N. & Gilbert, T. Desert Antelopes on the Brink: How Resilient is the Sahelo-Saharan Ecosystem? In *Antelope Conservation*: *From Diagnosis to Action* 253–279, 10.1002/9781118409572.ch13 (John Wiley & Sons, Ltd, 2016).

[59] Ochoa-Ochoa LM, Rodríguez P, Mora F, Flores-Villela O, Whittaker RJ (2012). Climate change and amphibian diversity patterns in Mexico. Biol. Conserv..

[60] McGill BJ (2007). Species abundance distributions: moving beyond single prediction theories to integration within an ecological framework. Ecol. Lett..

[61] Krebs, C. *The Ecological World View*. (CSIRO Publishing, 2008).

[62] Merow C (2014). What do we gain from simplicity versus complexity in species distribution models?. Ecography..

[63] Soultan A, Safi K (2017). The interplay of various sources of noise on reliability of species distribution models hinges on ecological specialisation. PLoS One.

[64] Bystriakova N, Peregrym M, Erkens RHJ, Bezsmertna O, Schneider H (2012). Sampling bias in geographic and environmental space and its effect on the predictive power of species distribution models. Syst. Biodivers..

[65] Hortal J, Jiménez-Valverde A, Gómez JF, Lobo JM, Baselga A (2008). Historical bias in biodiversity inventories affects the observed environmental niche of the species. Oikos.

[66] Elith J, Kearney M, Phillips S (2010). The art of modelling range-shifting species. Methods Ecol. Evol..

[67] Stohlgren TJ, Jarnevich CS, Esaias WE, Morisette JT (2011). Bounding species distribution models. Curr. Zool..

[68] Mokany K, Ferrier S (2011). Predicting impacts of climate change on biodiversity: a role for semi-mechanistic community-level modelling. Divers. Distrib..

[69] Ficetola GF, Pennati R, Manenti R (2013). Spatial segregation among age classes in cave salamanders: habitat selection or social interactions?. Popul. Ecol..

[70] Carvalho JC (2011). Biogeographic patterns of spiders in coastal dunes along a gradient of mediterraneity. Biodivers. Conserv..

[71] Ward D, Saltz D (1994). Forging at Different Spatial Scales: Dorcas Gazelles Foraging for Lilies in the Negev Desert. Ecology.

[72] Alqamy HEl, Din SBEl (2006). Contemporary status and distribution of gazelle species (*Gazella dorcas* and *Gazella leptoceros*) in Egypt. Zool. Middle East.

[73] Alqamy, H. Developing and Assessing a Population Monitoring Program for Dorcas Gazelle (*Gazella dorcas*) Using Distance Sampling in Southern Sinai, Egypt. (University of ST. Andrews, 2002).

[74] Osborn, D. J. & Helmy, I. *The contemporary land mammals of Egypt* (*including Sinai*), 10.5962/bhl.title.2801 (Fieldeiana Zoology, New Series, 1980).

[75] Durant SM (2017). The global decline of cheetah *Acinonyx jubatus* and what it means for conservation. Proc. Natl. Acad. Sci. USA.

[76] Stralberg D (2009). Re-Shuffling of Species with Climate Disruption: A No-Analog Future for California Birds?. PLoS One.

[77] Rouget M, Cowling RM, Pressey RL, Richardson DM (2003). Identifying spatial components of ecological and evolutionary processes for regional conservation planning in the Cape Floristic Region, South Africa. Divers. Distrib..

[78] Millar CI, Stephenson NL, Stephens SL (2007). Climate change and forests of the future: managing in the face of uncertainty. Ecol. Appl..

[79] Lawler JJ (2009). Climate Change Adaptation Strategies for Resource Management and Conservation Planning. Ann. N. Y. Acad. Sci..

[80] Peel MC, Finlayson BL, McMahon TA (2007). Updated world map of the Köppen-Geiger climate classification. Hydrol. Earth Syst. Sci..

[81] Wilson, D. E. & Reeder, D. M. (editors). *Mammal Species of the World*. *A Taxonomic and Geographic Reference*. (3rd ed. Johns Hopkins University Press, 2, 142pp. Available at http://www.press.jhu.edu, 2005).

[82] Shcheglovitova M, Anderson RP (2013). Estimating optimal complexity for ecological niche models: A jackknife approach for species with small sample sizes. Ecol. Modell..

[83] Radosavljevic A, Anderson RP (2014). Making better M axent models of species distributions: complexity, overfitting and evaluation. J. Biogeogr..

[84] Anderson RP, Raza A (2010). The effect of the extent of the study region on GIS models of species geographic distributions and estimates of niche evolution: preliminary tests with montane rodents (genus *Nephelomys*) in Venezuela. J. Biogeogr..

[85] Phillips SJ (2009). Sample selection bias and presence-only distribution models: implications for background and pseudo-absence data. Ecol. Appl..

[86] El-Gabbas A, Dormann CF (2018). Wrong, but useful: regional species distribution models may not be improved by range-wide data under biased sampling. Ecol. Evol..

[87] Kramer-Schadt S (2013). The importance of correcting for sampling bias in MaxEnt species distribution models. Divers. Distrib..

[88] Fourcade Y, Engler JO, Rödder D, Secondi J (2014). Mapping species distributions with MAXENT using a geographically biased sample of presence data: a performance assessment of methods for correcting sampling bias. PLoS One.

[89] Boria RA, Olson LE, Goodman SM, Anderson RP (2014). Spatial filtering to reduce sampling bias can improve the performance of ecological niche models. Ecol. Modell..

[90] Beck J, Böller M, Erhardt A, Schwanghart W (2014). Spatial bias in the GBIF database and its effect on modeling species’ geographic distributions. Ecol. Inform..

[91] Fourcade Y, Engler JO, Besnard AG, Rödder D, Secondi J (2013). Confronting expert-based and modelled distributions for species with uncertain conservation status: A case study from the corncrake (*Crex crex*). Biol. Conserv..

[92] Anderson MJ (2011). Navigating the multiple meanings of β diversity: a roadmap for the practicing ecologist. Ecol. Lett..

[93] Bradley BA (2016). Predicting abundance with presence-only models. Landsc. Ecol..

[94] Veloz SD (2009). Spatially autocorrelated sampling falsely inflates measures of accuracy for presence-only niche models. J. Biogeogr..

[95] Randin CF (2009). Climate change and plant distribution: local models predict high-elevation persistence. Glob. Chang. Biol..

[96] Graham MH (2003). Confronting multicollinearity in ecological multiple regression. Ecology.

[97] Townsend Peterson A, Papeş M, Eaton M (2007). Transferability and model evaluation in ecological niche modeling: a comparison of GARP and Maxent. Ecography..

[98] Quinn, G. P. & Keough, M. J. *Experimental design and data analysis for biologists*. (Cambridge University Press, 2002).

[99] Naimi, B. usdm: Uncertainty Analysis for Species Distribution Models. *R Packag*. *version 1*.*1*-*15*, https://cran.r-project.org/package=usdm (2015).

[100] Hijmans RJ, Cameron SE, Parra JL, Jones PG, Jarvis A (2005). Very high resolution interpolated climate surfaces for global land areas. Int. J. Climatol..

[101] Dormann CF (2013). Collinearity: a review of methods to deal with it and a simulation study evaluating their performance. Ecography..

[102] IPCC. *Climate change 2014*: *synthesis report*. *Contribution of working groups I*, *II and III to the fifth assessment report of the intergovernmental panel on climate change* (2014).

[103] Riahi K (2011). RCP 8.5—A scenario of comparatively high greenhouse gas emissions. Clim. Change.

[104] DiMiceli, C. M. *et al*. Annual Global Automated MODIS Vegetation Continuous Fields (MOD44B) at 250 m Spatial Resolution for Data Years Beginning Day 65, 2000–2010, Collection 5 Percent Tree Cover. (2011).

[105] Martin Y, Van Dyck H, Dendoncker N, Titeux N (2013). Testing instead of assuming the importance of land use change scenarios to model species distributions under climate change. Glob. Ecol. Biogeogr..

[106] Stanton JC, Pearson RG, Horning N, Ersts P, Reşit Akçakaya H (2012). Combining static and dynamic variables in species distribution models under climate change. Methods Ecol. Evol..

[107] Mesgaran MB, Cousens RD, Webber BL (2014). Here be dragons: a tool for quantifying novelty due to covariate range and correlation change when projecting species distribution models. Divers. Distrib..

[108] Araújo MB, New M (2007). Ensemble forecasting of species distributions. Trends Ecol. Evol..

[109] Marmion M, Parviainen M, Luoto M, Heikkinen RK, Thuiller W (2009). Evaluation of consensus methods in predictive species distribution modelling. Divers. Distrib..

[110] Phillips SJ, Anderson RP, Schapire RE (2006). Maximum entropy modeling of species geographic distributions. Ecol. Modell..

[111] Breiman L (2001). Random Forests. Mach. Learn..

[112] Elith J, Leathwick JR, Hastie T (2008). A working guide to boosted regression trees. J. Anim. Ecol..

[113] McCullagh, Peter & Nelder, J. *Generalized Linear Models*. (Chapman and Hall, 1989).

[114] Thuiller, W., Georges, D. & Engler, R. biomod2: Ensemble platform for species distribution modeling. *R Packag*. *version 3*.*3*-*13*/*r726*, https://r-forge.r-project.org/projects/biomod/ (2016).

[115] Belkhiria J (2016). Application of Species Distribution Modeling for Avian Influenza surveillance in the United States considering the North America Migratory Flyways. Sci. Rep..

[116] Hung C-M (2014). Drastic population fluctuations explain the rapid extinction of the passenger pigeon. Proc. Natl. Acad. Sci. USA.

[117] Warren DL, Wright AN, Seifert SN, Shaffer HB (2014). Incorporating model complexity and spatial sampling bias into ecological niche models of climate change risks faced by 90 California vertebrate species of concern. Divers. Distrib..

[118] Anderson RP, Gonzalez I (2011). Species-specific tuning increases robustness to sampling bias in models of species distributions: An implementation with Maxent. Ecol. Modell..

[119] Brown JL, Cameron A, Yoder AD, Vences M, Jarvis A (2014). A necessarily complex model to explain the biogeography of the amphibians and reptiles of Madagascar. Nat. Commun..

[120] Phillips SJ, Dudík M (2008). Modeling of species distributions with Maxent: new extensions and a comprehensive evaluation. Ecography..

[121] Phillips SJ (2008). Transferability, sample selection bias and background data in presence-only modelling: a response to Peterson *et al*. (2007). Ecography..

[122] Bagchi R (2018). Forecasting potential routes for movement of endemic birds among important sites for biodiversity in the Albertine Rift under projected climate change. Ecography..

[123] Baker DJ (2015). Assessing climate change impacts for vertebrate fauna across the West African protected area network using regionally appropriate climate projections. Divers. Distrib..

[124] Bagchi R (2013). Evaluating the effectiveness of conservation site networks under climate change: accounting for uncertainty. Glob. Chang. Biol..

[125] Fielding AH, Bell JF (1997). A review of methods for the assessment of prediction errors in conservation presence/absence models. Environ. Conserv..

[126] Allouche O, Tsoar A, Kadmon R (2006). Assessing the accuracy of species distribution models: prevalence, kappa and the true skill statistic (TSS). J. Appl. Ecol..

[127] Baker DJ, Hartley AJ, Butchart SHM, Willis SG (2016). Choice of baseline climate data impacts projected species’ responses to climate change. Glob. Chang. Biol..

[128] Buisson L, Thuiller W, Casajus N, Lek S, Grenouillet G (2010). Uncertainty in ensemble forecasting of species distribution. Glob. Chang. Biol..

[129] Goberville E, Beaugrand G, Hautekèete N-C, Piquot Y, Luczak C (2015). Uncertainties in the projection of species distributions related to general circulation models. Ecol. Evol..

[130] Cianfrani C, Broennimann O, Loy A, Guisan A (2018). More than range exposure: Global otter vulnerability to climate change. Biol. Conserv..

[131] Beale CM (2012). Incorporating uncertainty in predictive species distribution modelling. Philos. Trans. R. Soc. Lond. B. Biol. Sci..

[132] Barbet-Massin M, Thuiller W, Jiguet F (2010). How much do we overestimate future local extinction rates when restricting the range of occurrence data in climate suitability models?. Ecography..

[133] Jiang H (2016). Predicting the Potential Distribution of *Polygala tenuifolia Willd*. under Climate Change in China. PLoS One.

[134] Pineda E, Lobo JM (2009). Assessing the accuracy of species distribution models to predict amphibian species richness patterns. J. Anim. Ecol..

[135] González-Orozco CE (2014). Quantifying Phytogeographical Regions of Australia Using Geospatial Turnover in Species Composition. PLoS One.

[136] Oksanen, J. *et al*. vegan: Community Ecology Package. *R Packag*. *version 2*.*4*-*4*, https://CRAN.R-project.org/package=vegan (2017).

[137] IUCN. *IUCN Red List Categories and Criteria*: *Version 3*.*1* (IUCN, 2012).

[138] IUCN Standards and Petitions Subcommittee. *Guidelines for Using the IUCN Red List Categories and Criteria. Version 11* (2014).

