# Supplementary material for: Risk of biodiversity collapse under climate change in the Afro-Arabian region
Source: Sci Rep. 2019 Jan 30;9:955. doi: 10.1038/s41598-018-37851-6 (PMC6353965; doi:10.1038/s41598-018-37851-6)
Supplement: Supplementary file 1 — Supplementary information [file 41598_2018_37851_MOESM1_ESM.pdf]

Risk of biodiversity collapse under climate change in the Afro-Arabian region

Alaaeldin Soultan[1,2*], Martin Wikelski[1,2] and Kamran Safi[1,2]

[1.] Max Planck Institute for Ornithology, Department of Migration and Immuno-ecology, Am Obstberg 1, 78315 Radolfzell, Germany

[2.] University of Konstanz, Department of Biology, Universitätsstraße 10, 78464 Konstanz, Germany

[*]Corresponding author asoultan@orn.mpg.de

Supplementary Methods

Environmental sampling bias assessment

It is highly important to estimate whether sampling bias in geographical space induced bias in environmental space, and further, whether some environments are under-sampled. For this study we considered only species that have been comprehensively sampled over long periods of time (1960-2005) to ensure that species are at equilibrium with their environments. Therefore, we are confident that species occurrences used in our study provide a good estimate of the potential niches based on the fact that these occurrences were sampled along 50 years. As such, species have had time to sample the wide environmental conditions encompassed by the study region. Moreover, we computed kernel density estimates to assess the overlap between the distribution of our species occurrences and the distribution of the environments in the study area. We used the entire mammal dataset collected between 1960 and 2005 from the study area; this would provide a better estimation for under-sampled environments. The result shows large overlap between the distribution of species occurrences and the environments (Figure S1), which indicates that the environments are not underrepresented, and the sampled occurrences have good coverage of the study area.

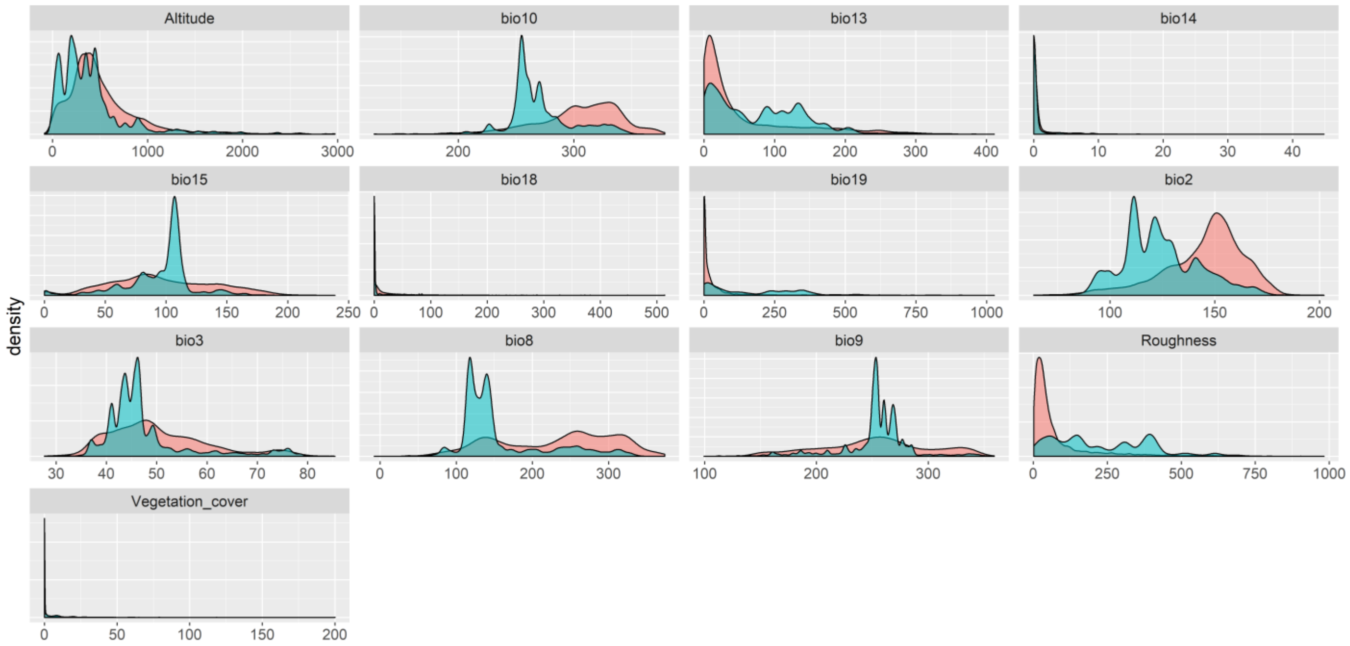

Figure S1: The density distribution for each environmental variable (red colour) overlapped with the density distribution of mammal's species (green colour) collected from the study area between 1960 and 2005

Supplementary Methods

Non-analogue environments and extrapolation

We identify the presence of non-analogue environments using the Extrapolation Detection tool (ExDet), a multivariate statistical tool based on the Mahalanobis distance, that measures the similarity of the temporal variables by accounting for deviation from the mean and correlation between variables (Mesgaran et al. 2014). ExDet identified two main regions within the study area with non-analogue environments (Figures S2 and S3). Therefore, the current distributions were projected onto the future climate scenarios using the 'clamping' option, which allows some extrapolation onto non-analogue environments in a constrained manner (Bateman et al. 2012). In practice, clamping option reduces the predictions by the absolute difference between the prediction with and without clamping (and setting the result at zero if the difference is negative), such that extrapolation is faded as predictions stray from known conditions (Bateman et al. 2012). We acknowledge that there is also another conservative approach, which completely avoids model extrapolation, by setting the value of a covariate that is beyond its training range to be zero (Stohlgren et al. 2011). However, following IUCN guidelines, we found this approach might not be the optimal as it truncates response curves and produces less reliable projections (Thuiller et al. 2003, Williams and Jackson 2007, Elith and Graham 2009, IUCN Standards and Petitions Subcommittee 2014).

References:

Bateman, B. L. et al. 2012. Biotic interactions influence the projected distribution of a specialist mammal under climate change. *Divers. Distrib*. 18: 861–872.

Elith, J. and Graham, C. H. 2009. Do they? How do they? WHY do they differ? On finding reasons for differing performances of species distribution models. *Ecography*. 32: 66–77.

IUCN Standards and Petitions Subcommittee 2014. Guidelines for Using the IUCN Red List Categories and Criteria. Version 11.

Mesgaran, M. B. et al. 2014. Here be dragons: a tool for quantifying novelty due to covariate range and correlation change when projecting species distribution models. *Divers. Distrib*. 20: 1147–1159.

Stohlgren, T. J. et al. 2011. Bounding species distribution models. *Curr. Zool*. 57: 642–647.

Thuiller, W. et al. 2003. Generalized models vs. classification tree analysis: Predicting spatial distributions of plant species at different scales. *J. Veg. Sci*. 14: 669–680.

Williams, J. W. and Jackson, S. T. 2007. Novel climates, no-analog communities, and ecological surprises. *Front. Ecol. Environ*. 5: 475–482.

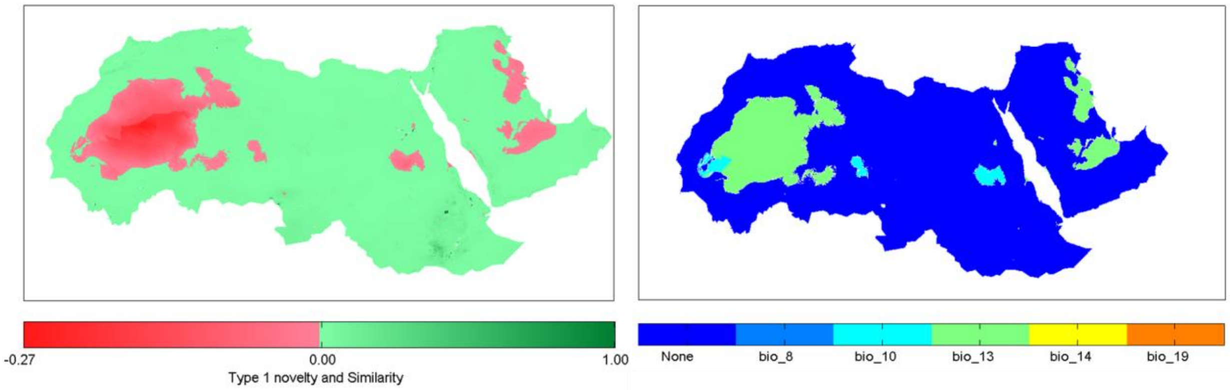

Figure S2: Availability of non-analogue environments between the current time and 2050. In left panel, Green are regions of similar environments (0 indicates maximum similarity), while Red, are regions with at least one variable outside of the univariate range (type 1 novelty). The more negative values the type 1 novelty are, the less similar the climates are in these regions. Right panel shows the spatial distribution of the most dissimilar covariate in terms of their contribution to both Type 1 novelty and Type 2 novelty. Blue areas have no covariate outside the coverage of calibration data.

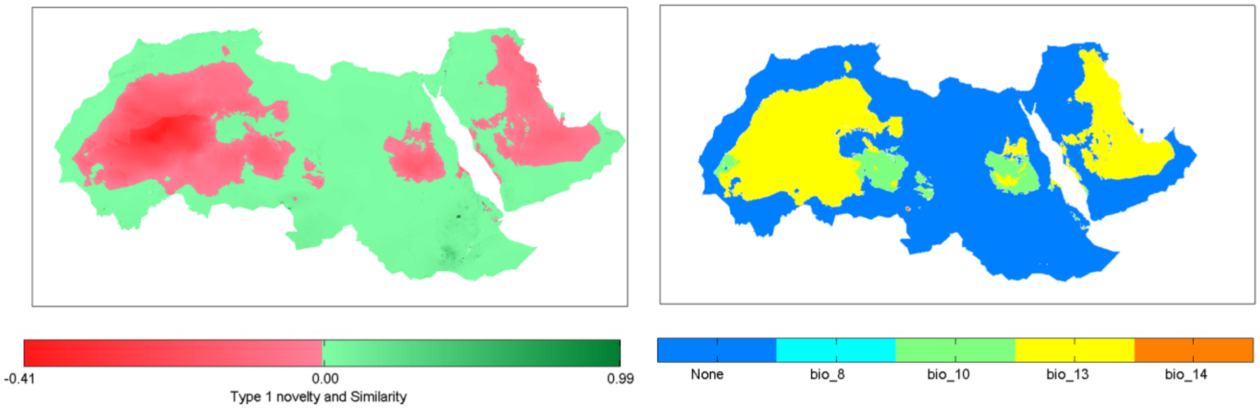

Figure S3: Availability of non-analogue environments between the current time and 2070. In left panel, Green are regions of similar environments (0 indicates maximum similarity), while Red, are regions with at least one variable outside of the univariate range (type 1 novelty). The more negative values the type 1 novelty are, the less similar the climates are in these regions. Right panel shows the spatial distribution of the most dissimilar covariate in terms of their contribution to both Type 1 novelty and Type 2 novelty. Blue areas have no covariate outside the coverage of calibration data.

Table S1: A list of the 107 endemic mammal species to the Afro-Arabian region. The "IUCN Status" column refers to the current threat category assigned to the corresponding species by IUCN. The "Endemism" column refers to the endemic species (its entire distribution range with the study area) and near endemic species (>70% of its distribution range within the study area).

Species	IUCN Status	Endemism
Acomys airensis	Least Concern	Endemic
Acomys cahirinus	Least Concern	Endemic
Acomys cineraceus	Least Concern	Endemic
Acomys mullah	Least Concern	Near endemic
Acomys russatus	Least Concern	Endemic
Addax nasomaculatus	Critically Endangered	Endemic
Allactaga euphratica	Near Threatened	Endemic
Allactaga tetradactyla	Vulnerable	Near endemic
Ammotragus lervia	Vulnerable	Endemic
Arabitragus jayakari	Endangered	Endemic
Asellia tridens	Least Concern	Endemic
Atelerix algirus	Least Concern	Near endemic
Atlantoxerus getulus	Least Concern	Endemic
Canis simensis	Endangered	Endemic
Capra nubiana	Vulnerable	Endemic
Crocidura aleksandrisi	Least Concern	Endemic
Crocidura arabica	Least Concern	Endemic
Crocidura baileyi	Endangered	Endemic
Crocidura bottegi	Data Deficient	Endemic
Crocidura bottegoides	Endangered	Endemic
Crocidura cinderella	Least Concern	Near endemic
Crocidura floweri	Data Deficient	Endemic
Crocidura fulvastra	Least Concern	Near endemic
Crocidura glassi	Vulnerable	Endemic
Crocidura lucina	Vulnerable	Endemic
Crocidura lusitania	Least Concern	Near endemic
Crocidura macmillani	Vulnerable	Endemic
Crocidura pasha	Least Concern	Endemic
Crocidura religiosa	Data Deficient	Endemic
Crocidura somalica	Least Concern	Endemic
Crocidura tarfayensis	Data Deficient	Endemic
Crocidura thalia	Least Concern	Endemic
Crocidura whitakeri	Least Concern	Endemic
Ctenodactylus gundi	Least Concern	Endemic
Ctenodactylus vali	Data Deficient	Endemic
Elephantulus rozeti	Least Concern	Endemic
Eliomys melanurus	Least Concern	Endemic
Eliomys munbyanus	Least Concern	Endemic
Eptesicus floweri	Least Concern	Endemic
Equus africanus	Critically Endangered	Endemic
Eudorcas albonotata	Least Concern	Endemic
Eudorcas rufifrons	Vulnerable	Near endemic
Eudorcas tilonura	Not Evaluated	Endemic
Felovia vae	Data Deficient	Endemic
Gazella cuvieri	Endangered	Endemic

Gazella dorcas	Vulnerable	Endemic
Gazella gazella	Vulnerable	Endemic
Gazella leptoceros	Endangered	Endemic
Genetta abyssinica	Least Concern	Endemic
Gerbillus amoenus	Least Concern	Endemic
Gerbillus andersoni	Least Concern	Endemic
Gerbillus bottai	Data Deficient	Endemic
Gerbillus campestris	Least Concern	Endemic
Gerbillus cheesmani	Least Concern	Endemic
Gerbillus dasyurus	Least Concern	Endemic
Gerbillus famulus	Least Concern	Endemic
Gerbillus gerbillus	Least Concern	Endemic
Gerbillus henleyi	Least Concern	Endemic
Gerbillus hoogstraali	Vulnerable	Endemic
Gerbillus latastei	Least Concern	Endemic
Gerbillus lowei	Data Deficient	Endemic
Gerbillus mackillingini	Least Concern	Endemic
Gerbillus maghrebi	Least Concern	Endemic
Gerbillus nancillus	Data Deficient	Endemic
Gerbillus nanus	Least Concern	Near endemic
Gerbillus nigeriae	Least Concern	Endemic
Gerbillus perpallidus	Least Concern	Endemic
Gerbillus poecilops	Least Concern	Endemic
Gerbillus pyramidum	Least Concern	Endemic
Gerbillus rosalinda	Least Concern	Endemic
Gerbillus rupicola	Least Concern	Endemic
Gerbillus simoni	Least Concern	Endemic
Gerbillus stigmonyx	Data Deficient	Endemic
Gerbillus tarabuli	Least Concern	Endemic
Gerbillus watersi	Least Concern	Endemic
Ictonyx libycus	Least Concern	Endemic
Jaculus jaculus	Least Concern	Endemic
Jaculus orientalis	Least Concern	Endemic
Kobus megaceros	Endangered	Endemic
Macaca sylvanus	Endangered	Endemic
Massoutiera mzabi	Least Concern	Endemic
Meriones crassus	Least Concern	Near endemic
Meriones rex	Least Concern	Endemic
Meriones sacramenti	Vulnerable	Endemic
Meriones shawi	Least Concern	Endemic
Mustela subpalmata	Least Concern	Endemic
Myotis punicus	Near Threatened	Endemic
Nanger dama	Critically Endangered	Endemic
Nanger soemmerringii	Vulnerable	Endemic
Oryx leucoryx	Vulnerable	Endemic
Pachyuromys duprasi	Least Concern	Endemic
Papio hamadryas	Least Concern	Endemic
Pipistrellus ariel	Data Deficient	Endemic
Plecotus christii	Data Deficient	Endemic
Psammomys obesus	Least Concern	Endemic

Psammomys vexillaris	Data Deficient	Endemic
Rhinopoma microphyllum	Least Concern	Near endemic
Sekeetamys calurus	Least Concern	Endemic
Spalax ehrenbergi	Data Deficient	Near endemic
Tachyoryctes macrocephalus	Endangered	Endemic
Taterillus arenarius	Least Concern	Endemic
Taterillus tranieri	Least Concern	Endemic
Theropithecus gelada	Least Concern	Endemic
Tragelaphus buxtoni	Endangered	Endemic
Vulpes pallida	Least Concern	Near endemic
Vulpes rueppellii	Least Concern	Endemic
Vulpes zerda	Least Concern	Endemic

Table S2: The mean and standard deviation of the evaluation metrics (AUC and TSS) used to evaluate SDM predictive performance for each species. Both AUC and TSS have been calculated from spatial-block cross-validation to maintain spatial independence between calibrating and evaluating data.

Species	AUC	Standard Deviation	TSS	Standard Deviation
Acomys airensis	0.94	0.05	0.89	0.11
Acomys cahirinus	0.87	0.01	0.74	0.02
Acomys cineraceus	0.87	0.03	0.75	0.06
Acomys mullah	0.95	0.06	0.91	0.13
Acomys russatus	0.91	0.03	0.82	0.07
Addax nasomaculatus	0.88	0.03	0.77	0.06
Allactaga euphratica	0.93	0.04	0.86	0.08
Allactaga tetradactyla	0.89	0.07	0.79	0.14
Ammotragus lervia	0.88	0.01	0.76	0.01
Arabitragus jayakari	0.91	0.03	0.83	0.07
Asellia tridens	0.86	0.03	0.73	0.06
Atelerix algirus	0.91	0.04	0.82	0.09
Atlantoxerus getulus	0.91	0.04	0.83	0.08
Canis simensis	0.83	0.15	0.67	0.31
Capra nubiana	0.89	0.02	0.79	0.04
Crocidura aleksandrisi	0.95	0.06	0.91	0.12
Crocidura arabicai	0.95	0.06	0.91	0.12
Crocidura baileyi	0.95	0.06	0.91	0.13
Crocidura bottegi	0.95	0.06	0.90	0.13
Crocidura bottegoides	0.78	0.22	0.57	0.44
Crocidura cinderella	0.92	0.03	0.85	0.07
Crocidura floweri	0.95	0.07	0.90	0.15
Crocidura fulvastra	0.89	0.01	0.78	0.03
Crocidura glassi	0.85	0.2	0.71	0.41
Crocidura lucina	0.83	0.25	0.66	0.51
Crocidura lusitania	0.94	0.05	0.88	0.11
Crocidura macmillani	0.95	0.06	0.91	0.13
Crocidura pasha	0.83	0.03	0.67	0.07
Crocidura religiosa	0.79	0.23	0.59	0.46
Crocidura somalica	0.86	0.01	0.73	0.03
Crocidura tarfayensis	0.91	0.03	0.82	0.06
Crocidura thalia	0.93	0.05	0.87	0.1

Species	AUC	Standard Deviation	TSS	Standard Deviation
Crocidura whitakeri	0.88	0.01	0.76	0.02
Ctenodactylus gundi	0.92	0.03	0.84	0.07
Ctenodactylus vali	0.95	0.06	0.91	0.12
Elephantulus rozeti	0.93	0.04	0.86	0.09
Eliomys melanurus	0.92	0.04	0.84	0.09
Eliomys munbyanus	0.91	0.03	0.83	0.07
Eptesicus floweri	0.95	0.06	0.9	0.12
Equus africanus	0.92	0.04	0.85	0.08
Eudorcas albonotata	0.91	0.04	0.83	0.08
Eudorcas rufifrons	0.89	0.02	0.81	0.04
Eudorcas tilonura	0.90	0.03	0.80	0.07
Felovia vae	0.92	0.04	0.85	0.09
Gazella cuvieri	0.90	0.05	0.8	0.1
Gazella dorcas	0.85	0.01	0.71	0.01
Gazella gazella	0.91	0.02	0.82	0.05
Gazella leptoceros	0.84	0.01	0.69	0.03
Genetta abyssinica	0.93	0.05	0.86	0.1
Gerbillus amoenus	0.88	0.02	0.76	0.04
Gerbillus andersoni	0.92	0.04	0.85	0.09
Gerbillus bottai	0.95	0.06	0.9	0.12
Gerbillus campestris	0.91	0.04	0.82	0.08
Gerbillus cheesmani	0.88	0.03	0.76	0.06
Gerbillus dasyurus	0.92	0.03	0.84	0.07
Gerbillus famulus	0.92	0.04	0.85	0.08
Gerbillus gerbillus	0.89	0.01	0.79	0.02
Gerbillus henleyi	0.89	0.03	0.79	0.06
Gerbillus hoogstraali	0.95	0.06	0.91	0.12
Gerbillus latastei	0.92	0.04	0.85	0.08
Gerbillus lowei	0.82	0.25	0.65	0.51
Gerbillus mackilligini	0.93	0.04	0.86	0.09
Gerbillus maghrebi	0.95	0.06	0.91	0.13
Gerbillus nancillus	0.90	0.03	0.80	0.06
Gerbillus nanus	0.78	0.01	0.57	0.02
Gerbillus nigeriae	0.91	0.03	0.82	0.07
Gerbillus perpallidus	0.80	0.23	0.60	0.46
Gerbillus poecilops	0.92	0.04	0.85	0.08

Species	AUC	Standard Deviation	TSS	Standard Deviation
Gerbillus pyramidum	0.88	0.01	0.77	0.03
Gerbillus rosalinda	0.92	0.04	0.84	0.08
Gerbillus rupicola	0.93	0.04	0.86	0.09
Gerbillus simoni	0.95	0.07	0.9	0.14
Gerbillus stigmonyx	0.90	0.04	0.81	0.09
Gerbillus tarabuli	0.87	0.05	0.74	0.1
Gerbillus watersi	0.93	0.04	0.86	0.08
Ictonyx libycus	0.91	0.03	0.82	0.06
Jaculus jaculus	0.83	0.01	0.66	0.01
Jaculus orientalis	0.91	0.03	0.83	0.07
Kobus megaceros	0.95	0.06	0.91	0.12
Macaca sylvanus	0.74	0.2	0.49	0.4
Massoutiera mzabi	0.91	0.03	0.83	0.06
Meriones crassus	0.87	0.01	0.74	0.02
Meriones rex	0.93	0.04	0.86	0.09
Meriones sacramenti	0.95	0.06	0.91	0.13
Meriones shawi	0.93	0.04	0.86	0.09
Mustela subpalmata	0.92	0.05	0.85	0.1
Myotis punicus	0.93	0.05	0.86	0.1
Nanger dama	0.83	0.02	0.66	0.05
Nanger soemmerringii	0.90	0.05	0.8	0.1
Oryx leucoryx	0.87	0.01	0.74	0.03
Pachyuromys duprasi	0.89	0.02	0.81	0.05
Papio hamadryas	0.91	0.03	0.83	0.07
Pipistrellus ariel	0.93	0.04	0.86	0.08
Plecotus christii	0.92	0.04	0.84	0.08
Psammomys obesus	0.90	0.02	0.81	0.05
Psammomys vexillaris	0.93	0.04	0.87	0.09
Rhinopoma microphyllum	0.92	0.04	0.85	0.08
Sekeetamys calurus	0.92	0.04	0.84	0.08
Spalax ehrenbergi	0.91	0.03	0.82	0.07
Tachyoryctes macrocephalus	0.83	0.25	0.66	0.51
Taterillus arenarius	0.95	0.06	0.91	0.12

Species	AUC	Standard Deviation	TSS	Standard Deviation
Taterillus tranieri	0.93	0.05	0.87	0.1
Theropithecus gelada	0.91	0.04	0.82	0.09
Tragelaphus buxtoni	0.79	0.23	0.59	0.46
Vulpes pallida	0.93	0.04	0.86	0.09
Vulpes rueppellii	0.87	0.01	0.75	0.01
Vulpes zerda	0.91	0.03	0.82	0.07

Table S3: Predicted temporal changes in the potential distribution ranges of endemic mammals in the Afro-Arabian region. “%UD” columns refer to the percentage change in the size of the distribution ranges based on the unlimited dispersal scenario. “UD” columns refer to the updated conservation status according to IUCN RedList A3c criterion. “%ND” columns refer to the percentage change in the size of the distribution ranges based on the no dispersal scenario. “ND” column refer to the updated conservation status according to IUCN RedList A3c criterion. “SDM algorithm” column refers to the model algorithms used to predicted distribution range. The negative number refers to the percentage loss in the projected distribution range of the corresponding species.

Species name	2050				2070				SDM Algorithm
	%UD	UD	% ND	ND	% UD	UD	%ND	ND	
Acomys airensis	> 100	LC	0.00	LC	> 100	LC	0.00	LC	MAXENT
Acomys cahirinus	-24.14	LC	-34.09	VU	-41.83	VU	-48.04	VU	MAXENT, GLM,GBM,RF
Acomys cineraceus	-9.07	LC	-10.03	LC	-9.51	LC	-10.45	LC	MAXENT
Acomys mullah	> 100	LC	-62.27	EN	> 100	LC	-71.17	EN	MAXENT
Acomys russatus	-58.98	EN	-60.71	EN	-69.10	EN	-70.29	EN	MAXENT, GLM,GBM,RF
Addax nasomaculatus	52.33	LC	-3.16	LC	66.83	LC	-2.23	LC	MAXENT, GLM
Allactaga euphratica	-18.67	LC	-23.17	LC	-22.68	LC	-31.52	VU	MAXENT, GLM
Allactaga tetradactyla	-100	EX	-100	EX	-100	EX	-100	EX	MAXENT, GLM
Ammotragus lervia	2.98	LC	-16.44	LC	7.64	LC	-15.09	LC	MAXENT, GLM,GBM
Arabitragus jayakari	> 100	LC	-5.15	LC	> 100	LC	-5.68	LC	MAXENT
Asellia tridens	-12.92	LC	-20.92	LC	-26.72	LC	-33.86	VU	MAXENT, GLM,GBM,RF
Atelerix algirus	-72.68	EN	-76.13	EN	-77.69	EN	-79.12	EN	MAXENT, GLM
Atlantoxerus getulus	-36.66	VU	-50.76	EN	-62.39	EN	-69.63	EN	MAXENT
Canis simensis	-33.02	VU	-33.83	VU	-49.66	VU	-50.14	EN	MAXENT, GLM
Capra nubiana	-32.12	VU	-42.97	VU	-46.37	VU	-53.89	EN	MAXENT, GLM,GBM,RF
Crocidura aleksandrisi	-100	EX	-100	EX	-100	EX	-100	EX	MAXENT
Crocidura arabicai	3.28	LC	-42.62	VU	76.23	LC	-21.31	LC	MAXENT
Crocidura baileyi	-36.10	VU	-53.17	EN	-46.83	VU	-52.68	EN	MAXENT
Crocidura bottegi	-94.87	CR	-100	EX	-100	EX	-100	EX	MAXENT
Crocidura bottegoides	-76.55	EN	-76.55	EN	-85.08	CR	-85.08	CR	MAXEN
Crocidura cinderella	-89.03	CR	-100	EX	-100	EX	-100	EX	MAXENT, GLM
Crocidura floweri	-100	EX	-100	EX	-100	EX	-100	EX	MAXENT
Crocidura fulvastra	-20.86	LC	-22.96	LC	-19.31	LC	-22.63	LC	MAXENT, GLM

Crocidura glassi	-81.55	CR	-81.55	CR	-100	EX	-100	EX	MAXENT
Crocidura lucina	-80.10	CR	-80.09	CR	-90.05	CR	-90.05	CR	MAXENT
Crocidura lusitania	-61.07	EN	-79.19	EN	-100	EX	-100	EX	MAXENT
Crocidura macmillani	-94.74	CR	-94.74	CR	-31.58	VU	-78.95	EN	MAXENT
Crocidura pasha	-14.66	LC	-17.31	LC	-15.17	LC	-17.76	LC	MAXENT
Crocidura religiosa	-99.17	EX	-99.17	EX	-99.83	EX	-99.83	EX	MAXENT, GLM
Crocidura somalica	19.55	LC	-4.14	LC	27.15	LC	-5.67	LC	MAXENT
Crocidura tarfayensis	-21.72	LC	-80.48	CR	-7.08	LC	-78.56	EN	MAXENT
Crocidura thalia	-77.70	EN	-77.70	EN	-92.07	CR	-92.07	CR	MAXENT, GLM
Crocidura whitakeri	-84.47	CR	-84.56	CR	-93.93	CR	-93.93	CR	MAXENT
Ctenodactylus gundi	-99.12	EX	-100	EX	-100	EX	-100	EX	MAXENT
Ctenodactylus vali	-100	EX	-100	EX	-100	EX	-100	EX	MAXENT
Elephantulus rozeti	-26.82	LC	-49.15	VU	-49.87	VU	-64.04	EN	MAXENT, GLM
Eliomys melanurus	-64.07	EN	-67.49	EN	-77.74	EN	-81.07	CR	MAXENT, GLM
Eliomys munbyanus	-81.57	CR	-81.57	CR	-92.90	CR	-92.90	CR	MAXENT, GLM
Eptesicus floweri	> 100	LC	-22.09	LC	> 100	LC	-26.10	LC	MAXENT
Equus africanus	-99.51	EX	-100	EX	-100	EX	-100	EX	MAXENT
Eudorcas albonotata	9.87	LC	-26.85	LC	39.57	LC	-23.68	LC	MAXENT, GLM
Eudorcas rufifrons	-0.06	LC	-28.88	LC	-14.43	LC	-41.17	VU	MAXENT, GLM
Eudorcas tilonura	-39.43	VU	-48.26	VU	-26.56	LC	-40.48	VU	MAXENT, GLM
Felovia vae	10.74	LC	-36.96	VU	-18.70	LC	-50.77	EN	MAXENT, GLM
Gazella cuvieri	-74.61	EN	-75.34	EN	-72.14	EN	-74.07	EN	MAXENT, GLM
Gazella dorcas	-40.58	VU	-44.25	VU	-60.84	EN	-61.57	EN	MAXENT, GLM,GBM,RF
Gazella gazella	-19.31	LC	-28.48	LC	-27.27	LC	-35.49	VU	MAXENT, GLM,GBM,RF
Gazella leptoceros	-76.46	EN	-76.50	EN	-91.77	CR	-91.85	CR	MAXENT, GLM,GBM
Genetta abyssinica	32.31	LC	-55.38	EN	24.62	LC	-63.08	EN	MAXENT
gerbillus amoenus	-81.34	CR	-81.41	CR	-91.01	CR	-91.01	CR	MAXENT, GLM
Gerbillus andersoni	-91.84	CR	-93.87	CR	-98.43	EX	-99.59	EX	MAXENT, GLM,GBM,RF
Gerbillus bottai	-100	EX	-100	EX	-100	EX	-100	EX	MAXENT
Gerbillus campestris	-4.61	LC	-90.98	CR	-47.69	VU	-94.34	CR	MAXENT, GLM
Gerbillus cheesmani	22.72	LC	-17.87	LC	13.20	LC	-26.89	LC	MAXENT, GLM

Gerbillus dasyurus	-53.53	EN	-61.55	EN	-42.45	VU	-57.73	EN	MAXENT, GLM,GBM,RF
Gerbillus famulus	-99.27	EX	-100	EX	-98.73	EX	-99.64	EX	MAXENT, GLM
Gerbillus gerbillus	-51.83	EN	-52.54	EN	-73.67	EN	-73.97	EN	MAXENT, GLM,GBM,RF
Gerbillus henleyi	-50.83	EN	-51.82	EN	-69.35	EN	-69.49	EN	MAXENT, GLM,GBM,RF
Gerbillus hoogstraali	-100	EX	-100	EX	-100	EX	-100	EX	MAXENT, GLM
Gerbillus latastei	-12.15	LC	-31.34	VU	-36.37	VU	-44.37	VU	MAXENT, GLM
Gerbillus lowei	-100	EX	-100	EX	-100	EX	-100	EX	MAXENT
Gerbillus mackilligini	> 100	LC	-27.32	LC	39.35	LC	-47.00	VU	MAXENT, GLM
Gerbillus maghrebi	-100	EX	-100	EX	-100	EX	-100	EX	MAXENT
Gerbillus nancillus	8.93	LC	-34.40	VU	4.00	LC	-37.22	VU	MAXENT
Gerbillus nanus	45.27	LC	-3.97	LC	58.28	LC	-3.71	LC	MAXENT, GLM,GBM,RF
Gerbillus nigeriae	-25.74	LC	-46.06	LC	-38.78	VU	-55.47	EN	MAXENT, GLM
Gerbillus perpallidus	-100	EX	-100	EX	-100	EX	-100	EX	MAXENT, GLM
Gerbillus poecilops	-65.69	EN	-91.18	CR	-46.36	VU	-89.08	CR	MAXENT, GLM
Gerbillus pyramidum	-53.42	EN	-53.73	EN	-64.17	EN	-66.51	EN	MAXENT, GLM,GBM,RF
Gerbillus rosalinda	-100	EX	-100	EX	-100	EX	-100	EX	MAXENT, GLM
Gerbillus rupicola	-21.77	LC	-27.76	LC	-46.65	VU	-49.04	VU	MAXENT, GLM
Gerbillus simoni	-97.40	CR	-97.40	CR	-99.57	EX	-99.57	EX	MAXENT
Gerbillus stigmonyx	-28.87	LC	-41.05	VU	-31.50	VU	-48.76	VU	MAXENT, GLM
Gerbillus tarabuli	-64.87	EN	-76.66	EN	-56.80	EN	-77.82	EN	MAXENT, GLM,GBM,RF
Gerbillus watersi	-53.65	EN	-70.53	EN	-63.70	EN	-82.66	CR	MAXENT, GLM
Ictonyx libycus	-35.31	VU	-37.43	VU	-46.17	VU	-47.81	VU	MAXENT, GLM,GBM,RF
Jaculus jaculus	-54.60	EN	-55.23	EN	-66.87	EN	-67.57	EN	MAXENT, GLM,GBM,RF
Jaculus orientalis	-80.54	CR	-81.22	CR	-95.78	CR	-95.92	CR	MAXENT, GLM,GBM,RF
Kobus megaceros	16.07	LC	-66.07	EN	-60.71	EN	-71.43	EN	MAXENT
Macaca sylvanus	-3.23	LC	-17.20	LC	-5.92	LC	-24.08	LC	MAXENT, GLM
Massoutiera mzabi	> 100	LC	-3.87	LC	> 100	LC	-5.43	LC	MAXENT, GLM,GBM
Meriones crassus	-8.30	LC	-26.92	LC	-21.31	LC	-36.66	VU	MAXENT, GLM,GBM,RF

Meriones rex	> 100	LC	-7.12	LC	381.74	LC	-5.04	LC	MAXENT, GLM
Meriones sacramenti	-100	EX	-100	EX	-100	EX	-100	EX	MAXENT
Meriones shawi	-44.46	VU	-56.51	EN	-66.60	EN	-73.34	EN	MAXENT, GLM
Mustela subpalmata	1.83	LC	-8.14	LC	0.97	LC	-8.66	LC	MAXENT, GLM
Myotis punicus	-20.62	LC	-25.04	LC	-27.28	LC	-32.44	VU	MAXENT, GLM,GBM
Nanger dama	-81.82	CR	-82.14	CR	-91.54	CR	-91.79	CR	MAXENT, GLM
Nanger soemmerringii	69.84	LC	-11.15	LC	> 100	LC	-3.23	LC	MAXENT, GLM
Oryx leucoryx	5.59	LC	-10.26	LC	12.45	LC	-13.01	LC	MAXENT, GLM
Pachyuromys duprasi	-71.22	EN	-72.70	EN	-82.02	CR	-84.11	CR	MAXENT, GLM,GBM
papio hamadryas	52.03	LC	-3.46	LC	> 100	LC	-2.27	LC	MAXENT, GLM,GBM
Pipistrellus ariel	-71.38	EN	-73.82	EN	-86.13	CR	-89.25	CR	MAXENT, GLM
Plecotus christii	-75.93	EN	-76.04	EN	-87.04	CR	-87.19	CR	MAXENT, GLM,GBM,RF
Psammomys obesus	-25.01	LC	-30.86	VU	-34.42	VU	-39.37	VU	MAXENT, GLM,GBM,RF
Psammomys vexillaris	-38.96	VU	-44.60	VU	-70.79	EN	-75.98	EN	MAXENT
Rhinopoma microphyllum	-40.64	VU	-43.16	VU	-46.65	VU	-49.35	VU	MAXENT, GLM,GBM,RF
Sekeetamys calurus	-46.86	VU	-47.99	VU	-61.94	EN	-62.53	EN	MAXENT, GLM,GBM
Spalax ehrenbergi	-45.77	VU	-55.37	EN	-65.59	EN	-72.41	EN	MAXENT, GLM,GBM
Tachyoryctes macrocephalus	-93.98	CR	-93.98	CR	-100	EX	-100	EX	MAXENT, GLM,GBM
Taterillus arenarius	-29.17	LC	-39.20	VU	-45.65	VU	-51.82	EN	MAXENT
Taterillus tranieri	9.89	LC	-42.64	VU	-47.90	VU	-75.83	EN	MAXENT, GLM
Theropithecus gelada	12.61	LC	-13.50	LC	16.55	LC	-13.05	LC	MAXENT, GLM
Tragelaphus buxtoni	-85.02	CR	-85.02	CR	-89.83	CR	-89.83	CR	MAXENT, GLM
Vulpes pallida	> 100	LC	-9.58	LC	> 100	LC	-11.36	LC	MAXENT, GLM
Vulpes rueppellii	-38.79	VU	-42.34	VU	-56.35	EN	-59.15	EN	MAXENT, GLM,GBM,RF
Vulpes zerda	-29.65	LC	-40.57	VU	-41.33	VU	-51.76	EN	MAXENT, GLM,GBM,RF

Table S4: List of the species data source

Alemayehu, K. et al. 2012. Effects of habitat loss and limitation on effective population size and inbreeding rates of Walia ibex (*Capra walie*) in Ethiopia. - Afr. J. Ecol. 50: 125–130.
ACR 2015. African Chiroptera Report. 2015. African Bats, African Chiroptera Project,Pretoria, i–xix + 7001.
Beudels, R.C., Devillers, P., Lafontaine, R.M., Devillers-Terschuren, J. & Beudels, M.O. (eds) (2005) Sahelo-Saharan antelopes: status and perspectives. Report on the conservation status of the six Sahelo-Saharan antelopes, CMS SSA Concerted Action, 2nd edn. *CMS Technical Series Publication No. 11*. UNEP/CMS Secretariat, Bonn.
BioMap Egypt. - http://www.biomapegypt.org/biodiversity/
Brito, J. C. et al. 2010. Data on the distribution of mammals from Mauritania, West Africa. - *Mammalia* 74: 449–455.
East, R. 1990. Antelopes - Global Survey and Regional Action Plan: Part 3 - West and Central Africa.
East, R. 1999. African Antelope Database 1998. *IUCN/SSC Antelope Specialist Group*.
Environment and Protected Areas Authority (EPAA) 2003. Fourth International Conservation Workshop for the Threatened Fauna of Arabia. BCEAW/EPAA; Sharjah, UAE.
GBIF 2016. Global Biodiversity Information Facility. - http://www.gbif.org/
Gecchele, L. V. et al. 2017. A pilot study to survey the carnivore community in the hyper-arid environment of South Sinai mountains. - *J. Arid Environ*. 141: 16–24.
Harrison, D. L. and Bates, P. J. J. 1991. The Mammals of Arabia. - Sevenoaks, Kent, England : Harrison Zoological Museum.
HATOUGH-BOURAN, A. 1990. The burrowing habits of desertic Rodents *Jaculus jaculus* and *Gerbillus dasyurus* in the Shaumari Reserve in Jordan. - Mammalia 54: 341–360.
IUCN/SSC 2012. Regional Conservation Strategy for the Cheetah and African Wild Dog in Western, Central and Northern Africa.
Kingdon, J. et al. 2013. Mammals of Africa (6 volumes). - *Bloomsbury Publishing*, London, United Kingdom.
Mallon, D. P. and Kingswood, S. C. 2001. Antelopes. Part 4: North Africa, the Middle East, and Asia. Global Survey and Regional Action Plans.
Mohammad Adnan Abu Baker and Zuhair Amr 2003. A morphometric and taxonomic revision of the genus Gerbillus (Mammalia, Rodentia, Gerbillidae) in Jordan with notes on its current distribution. - Zool. Abhandlungen 53: 177–204.
Soultan, A. et al. 2016. Recent observation for leopard *Panthera pardus* in Egypt. - Mammalia 0: 16–18.
YALDEN, D. W. 1988. Small mammals of the Bale Mountains, Ethiopia. - Afr. J. Ecol. 26: 281–294.
YALDEN, D. W. and LARGEN, M. J. 1992. The endemic mammals of Ethiopia. - Mamm. Rev. 22: 115–150.

Table S5: List of the environmental variables, climatic and non-climatic, and their sources. Only uncorrelated variables with Variance Inflation Factor (VIF) value less than 10 (corresponding to "yes" in this table) used for prediction.

Variable	Source	Used for Modeling
BIO1 = Annual Mean Temperature	http://www.worldclim.org/	No
BIO2 = Mean Diurnal Range (Mean of monthly (max temp - min temp))		Yes
BIO3 = Isothermality (BIO2/BIO7) (* 100)		Yes
BIO4 = Temperature Seasonality (standard deviation *100)		No
BIO5 = Max Temperature of Warmest Month		No
BIO6 = Min Temperature of Coldest Month		No
BIO7 = Temperature Annual Range (BIO5-BIO6)		No
BIO8 = Mean Temperature of Wettest Quarter		Yes
BIO9 = Mean Temperature of Driest Quarter		Yes
BIO10 = Mean Temperature of Warmest Quarter		Yes
BIO11 = Mean Temperature of Coldest Quarter		No
BIO12 = Annual Precipitation		No
BIO13 = Precipitation of Wettest Month		Yes
BIO14 = Precipitation of Driest Month		Yes
BIO15 = Precipitation Seasonality (Coefficient of Variation)		Yes
BIO16 = Precipitation of Wettest Quarter		No
BIO17 = Precipitation of Driest Quarter		No
BIO18 = Precipitation of Warmest Quarter		Yes
BIO19 = Precipitation of Coldest Quarter		Yes
Altitude		Yes
Roughness		Yes
Global Aridity Index	http://www.cgiar-csi.org/	No
EVT - Annual mean evapo-transpiration	http://earlywarning.usgs.gov	No
NPP - Net Primary Production	http://neo.sci.gsfc.nasa.gov	No
PET - Global Potential Evapo-Transpiration	http://www.cgiar-csi.org/	No
VCF - Vegetation continuous field	http://glcf.umd.edu/data/vcf/	Yes
Future emission scenario (RCP 8.5)	http://www.worldclim.org/	Yes

Table S6: The final set of predictors used for model calibration at the current and projection at the future (2050 and 2070) after calculating the collinearity between the predictors based on the Variance Inflation Factor (VIF) and removed the predictors with VIF value greater than 10.

Variables	Current	2050	2070
Altitude	2.70	2.43	2.20
bio_10	7.50	6.77	6.49
bio_13	3.89	4.04	3.76
bio_14	2.06	1.74	1.74
bio_15	1.99	2.24	2.19
bio_18	4.33	3.19	3.48
bio_19	1.81	1.99	1.99
bio_2	1.82	1.64	1.51
bio_3	4.76	5.07	5.30
bio_8	5.29	4.56	4.34
bio_9	2.38	2.19	2.29
Roughness	1.90	1.84	1.77
Vegetation field	1.21	1.26	1.26

Table S7: Number of species occurrences after spatial filtering and the contribution of the environmental variables used to identify species potential distribution. The second column shows the number of species occurrences used for prediction. The third to the last columns are the variables used for each species and the number refers to the percentage of variables contribution to model gain. Only variables with contribution greater than 5% to model gain retaining for the final model calibration and presented in this table.

Species	Occurrence Number	Altitude	Roughness	VCF - Vegetation continuous field	BIO2 = Mean Diurnal Range	BIO3 = Isothermality	BIO8 = Mean Temperature of Wettest Quarter	BIO9 = Mean Temperature of Driest Quarter	BIO10 = Mean Temperature of Warmest Quarter	BIO13 = Precipitation of Wettest Month	BIO14 = Precipitation of Driest Month	BIO15 = Precipitation Seasonality	BIO18 = Precipitation of Warmest Quarter	BIO19 = Precipitation of Coldest Quarter
Acomys airensis	5						11.35		26.48		8.77	27.99		20.27
Acomys cahirinus	257	5.77			9.81	12.69	24.01	10.45				14.56	5.77	9.08
Acomys cineraceus	14					5.2			12.58	72.90				
Acomys mullah	6				7.55		46.89				21.57	14.74		
Acomys russatus	107					22.34		12.58	22.82		6.05	14.71		7.84
Addax nasomaculatus	19								12.29			10.40		69.50
Allactaga euphratica	19								16.46	12.48		65.70		
Allactaga tetradactyla	16	22.83							38.45	22.87	5.16		9.96	
Ammotragus lervia	77	5.30			15.53				30.67	36.26				
Arabitragus jayakari	13			22.48	35.26					22.78		10.58		
Asellia tridens	183	25.55	24.59	14.92			12.15	6.70						6.42
Atelerix algirus	24				12.58		66.25					7.05		
Atlantoxerus getulus	18	14.19	7.09				41.88		5.35			12.58		
Canis simensis	35	6.19	8.20			35.48	5.58	11.33	7.85	9.07				
Capra nubiana	123	8	14.84		28.52		20.00	7.27			5.08		12.50	
Crocidura aleksandrisi	10					7.31	12.80		39.22		7.24		16.15	
Crocidura arabicai	6			9.00	44.64				12.29		12.58			
Crocidura baileyi	6	22.87			12.58		7.24			40.49				
Crocidura bottegi	10					62.60	5.02		27.86					
Crocidura bottegoides	8					18.53		5.33	59.33			8.13		
Crocidura cinderella	20	27.42									6.48	33.48		23.27
Crocidura floweri	5	47.52			11.58				14.25				12.23	

Species	Occurrence Number	Altitude	Roughness	VCF - Vegetation continuous field	BIO2 = Mean Diurnal Range	BIO3 = Isothermality	BIO8 = Mean Temperature of Wettest Quarter	BIO9 = Mean Temperature of Driest Quarter	BIO10 = Mean Temperature of Warmest Quarter	BIO13 = Precipitation of Wettest Month	BIO14 = Precipitation of Driest Month	BIO15 = Precipitation Seasonality	BIO18 = Precipitation of Warmest Quarter	BIO19 = Precipitation of Coldest Quarter
Crocidura fulvastra	11	7.95				12.51				58.01	20.95			
Crocidura glassi	5					20.41					46.01		6.66	16.57
Crocidura lucina	7					6.65			74.88	4.93				
Crocidura lusitania	10		8.2		49.38					16.67			12.48	
Crocidura macmillani	5					83.00				6.1	7.3			
Crocidura pasha	6	31.26				7.24			46.74				12.58	
Crocidura religiosa	14	45.13			12.08					15.63			13.94	
Crocidura somalica	7		9.48			24.45			5.58				62.23	
Crocidura tarfayensis	8	15.29				9.58			24.71	45.00				
Crocidura thalia	19					29.76			38.03		6.73	8.19		
Crocidura whitakeri	8	50.99				17.29			21.30				13.38	
Ctenodactylus gundi	11			11.44		43.88			25.35			14.18		
Ctenodactylus vali	9			15.83		25.45	45.55						7.58	
Elephantulus rozeti	40		5.08				16.88		14.22	16.48		21.88	8.13	7.42
Eliomys melanurus	45				19.48	5.34	34.17					10.08	10.28	
Eliomys munbyanus	23								63.29			11.64	18.53	
Eptesicus floweri	7			5.12			10.50					26.25		44.62
Equus africanus	10	10.28									16.87	57.49	9.28	
Eudorcas albonotata	20	13.10			5.75	36.96								32.19
Eudorcas rufifrons	45				23.93				8.03	28.36	8.52	6.89	11.15	
Eudorcas tilonura	14	13.06			5.01	9.02					65.53			
Felovia vae	44		12.79			5.93	55.42		7.27					
Gazella cuvieri	32	39.29							12.66			12.97	13.35	
Gazella dorcas	178	17.38			19.19	12.94				13.85	6.24	9.32		13.30
Gazella gazella	219				7.76			16.82	8.96	12.85		21.63	5.18	17.84
Gazella leptoceros	55	16.39			12.77			6.97	40.31	13.22			6.88	
Genetta abyssinica	7		9.84	15.55		7.24			9.58				38.37	
Gerbillus amoenus	23								58.14	30.55	7.86			
Gerbillus andersoni	110	5.51				10.59	8.52		30.12		6.97	22.46		
Gerbillus bottai	10		8.48			31.91		17.17						38.60

Species	Occurrence Number	Altitude	Roughness	VCF - Vegetation continuous field	BIO2 = Mean Diurnal Range	BIO3 = Isothermality	BIO8 = Mean Temperature of Wettest Quarter	BIO9 = Mean Temperature of Driest Quarter	BIO10 = Mean Temperature of Warmest Quarter	BIO13 = Precipitation of Wettest Month	BIO14 = Precipitation of Driest Month	BIO15 = Precipitation Seasonality	BIO18 = Precipitation of Warmest Quarter	BIO19 = Precipitation of Coldest Quarter
Gerbillus campestris	23	6.58							15.43		14.24	21.25	28.37	10.90
Gerbillus cheesmani	67								6.48	22.59		8.64	8.47	42.86
Gerbillus dasyurus	93				52.14	5.61				25.78		6.28		
Gerbillus famulus	11					31.62					6.49	11.43		35.19
Gerbillus gerbillus	292	33.18					17.09	8.14				17.46	7.22	
Gerbillus henleyi	97	18.36			26.97				13.61				15.14	
Gerbillus hoogstraali	11				10.08				53.43		27.98			
Gerbillus latastei	28	20.32					6.19		7.94	10.95			7.11	43.90
Gerbillus lowei	10				51.47				27.04					14.50
Gerbillus mackilligini	18	5.53								19.26	38.84		32.27	
Gerbillus maghrebi	10					5.69			62.64				5.60	19.68
Gerbillus nancillus	9	40.26			9.5				40.50				7.24	
Gerbillus nanus	111						13.54	13.76		21.24		7.15	12.03	20.59
Gerbillus nigeriae	19	37.54							6.87	38.15	17.44			
Gerbillus perpallidus	31	31.41							34.59	9.57	10.97		5.35	
Gerbillus poecilops	13				27.43	27.49				7.51		17.16		11.46
Gerbillus pyramidum	188	48.21			10.15	11.44			5.11				6.98	
Gerbillus rosalinda	20		6.22			21.27			17.36	5.53				37.40
Gerbillus rupicola	21	19.60	12.58		7.02	7.68			8.69	18.03		11.06		12.93
Gerbillus simoni	6	25.73			14.01	6.63			41.10		10.66			
Gerbillus stigmonyx	20		10.62			41.80						17.70		13.39
Gerbillus tarabuli	73	12.51			5.78	10.52			27.23	8.41	10.83	23.03		
Gerbillus watersi	21					31.52	10.41	5.64		32.00				13.13
Ictonyx libycus	13	19.66			7.17					5.56			54.55	7.66
Jaculus jaculus	279	18.79			12.82		13.12		13.02	14.91			16.30	
Jaculus orientalis	82	2.15				9.31	6.44		51.90	8.66		8.23		
Kobus megaceros	5		34.21	5.29		8.05			7.24				6.49	
Macaca sylvanus	22	61.89							8.11			6.03	7.24	
Massoutiera mzabi	52	20.76			9.7	34.28								25.78

Species	Occurrence Number	Altitude	Roughness	VCF - Vegetation continuous field	BIO2 = Mean Diurnal Range	BIO3 = Isothermality	BIO8 = Mean Temperature of Wettest Quarter	BIO9 = Mean Temperature of Driest Quarter	BIO10 = Mean Temperature of Warmest Quarter	BIO13 = Precipitation of Wettest Month	BIO14 = Precipitation of Driest Month	BIO15 = Precipitation Seasonality	BIO18 = Precipitation of Warmest Quarter	BIO19 = Precipitation of Coldest Quarter
Meriones crassus	183	10.78				12.93		16.65		14.85		21.44		9.22
Meriones rex	23	44.57			21.65	6.34				7.56			8.77	
Meriones sacramenti	9				35.46		11.39		20.63				21.85	
Meriones shawi	55	5.58				25.07	9.54		29.20	10.01		7.97		
Mustela subpalmata	20	58.11				11.5			7.24		10.56			
Myotis punicus	58						5.74		25.04	8.42	13.41		11.92	21.76
Nanger dama	21	15.85			31.87							12.58	29.93	
Nanger soemmerringii	37	7.67				43.92			11.36					16.93
Oryx leucoryx	44	15.19								8.51	27.90	46.66		
Pachyuromys duprasi	60	5.95			7.48			7.30	33.81	26.69				
Papio hamadryas	56	21.12			4.79	7.38			10.56	14.52		5.87		29.24
Pipistrellus ariel	18		7.17		41.70					33.53		5.25		
Plecotus christii	68		10.29	9.96			8.41	8.96	12.94	23.67			11.62	
Psammomys obesus	213	12.25			15.12	18.68	9.09	9.98	10.38	5.83			8.70	
Psammomys vexillaris	5	5.12			5.3	65.11			15.4					
Rhinopoma microphyllum	90	10.26		21.83					16.95	13.84	9.39		12.50	
Sekeetamys calurus	61	9.93				10.01			11.68	34.36	5.65	17.89		5.43
Spalax ehrenbergi	34	14.29			8.40					32.83	19.61		16.23	
Tachyoryctes macrocephalus	33				12.57				58.83	9.95				

Species	Occurrence Number	Altitude	Roughness	VCF - Vegetation continuous field	BIO2 = Mean Diurnal Range	BIO3 = Isothermality	BIO8 = Mean Temperature of Wettest Quarter	BIO9 = Mean Temperature of Driest Quarter	BIO10 = Mean Temperature of Warmest Quarter	BIO13 = Precipitation of Wettest Month	BIO14 = Precipitation of Driest Month	BIO15 = Precipitation Seasonality	BIO18 = Precipitation of Warmest Quarter	BIO19 = Precipitation of Coldest Quarter
Taterillus arenarius	6	36.47								17.39	13.73	26.27	5.62	
Taterillus tranieri	21	38.09								5.50		31.67		16.40
Theropithecus gelada	24	31.50			5.61	31.39				7.22		6.99		
Tragelaphus buxtoni	23	7.84				15.11			36.45			9.46	10.15	6.34
Vulpes pallida	30	35.77							5.82	33.86	11.22	5.71		
Vulpes rueppellii	173	9.37	13.09		7.88	13.09							44.91	
Vulpes zerda	74	38.65	7.48					5.35		11.27			15.63	5.35
